# TDRD5 binds piRNA precursors and selectively enhances pachytene piRNA processing in mice

**DOI:** 10.1038/s41467-017-02622-w

**Published:** 2018-01-09

**Authors:** Deqiang Ding, Jiali Liu, Uros Midic, Yingjie Wu, Kunzhe Dong, Ashley Melnick, Keith E. Latham, Chen Chen

**Affiliations:** 1 Department of Animal Science, Michigan State University, East Lansing, MI 48824 USA; 2 State Key Laboratory of Agrobiotechnology, College of Biological Sciences, China Agricultural University, Beijing, 100193 China; 3 College of Animal Science and Technology, China Agricultural University, Beijing, 100193 China; 4 USDA, Agricultural Research Service, Avian Disease and Oncology Laboratory, East Lansing, MI 48823 USA; 5 Reproductive and Developmental Sciences Program, Michigan State University, East Lansing, MI 48824 USA; 6 Department of Obstetrics, Gynecology and Reproductive Biology, Michigan State University, Grand Rapids, MI 49503 USA

## Abstract

Pachytene piRNAs are the most abundant piRNAs in mammalian adult testes. They are generated from long precursor transcripts by the primary piRNA biogenesis pathway but the factors involved in pachytene piRNA precursors processing are poorly understood. Here we show that the Tudor domain-containing 5 (TDRD5) protein is essential for pachytene piRNA biogenesis in mice. Conditional inactivation of TDRD5 in mouse postnatal germ cells reveals that TDRD5 selectively regulates the production of pachytene piRNAs from abundant piRNA-producing precursors, with little effect on low-abundant piRNAs. Unexpectedly, TDRD5 is not required for the 5′ end processing of the precursors, but is crucial for promoting production of piRNAs from the other regions of the transcript. Furthermore, we show that TDRD5 is an RNA-binding protein directly associating with piRNA precursors. These observations establish TDRD5 as a piRNA biogenesis factor and reveal two genetically separable steps at the start of pachytene piRNA processing.

## Abstract (web summary)

Pachytene piRNAs are abundant piRNAs in mammalian adult testes but their biogenesis pathway is not fully understood. Here, the authors identify TDRD5 as a piRNA biogenesis factor in mice, showing that it binds piRNA precursors and promotes pachytene piRNA production from specific transcript regions.

## Introduction

Maintaining germline genome integrity and RNA homeostasis is essential for gametogenesis. During mammalian spermatogenesis, PIWI-interacting RNAs (piRNAs), which comprise a class of germ cell-specific small non-coding RNAs, play a crucial role in silencing transposons and protecting the germline genome^1–7^. piRNAs also regulate spermatogenesis-associated RNAs, and are essential for the production of functional sperm^8–11^. The impairment of the piRNA pathway often results in transposon upregulation, spermatogenic arrest, and male infertility^12–14^.

piRNAs are produced by cleavages of precursor RNAs through primary processing and secondary amplification, and exert their function through associated PIWI proteins^12,15^. During mouse spermatogenesis, two distinct populations of piRNAs become associated with the PIWI proteins (MILI, MIWI, and MIWI2) at two different developmental stages. Embryonic/perinatal male germ cells produce a population of transposon sequence-rich piRNAs (TE piRNAs or prepachytene piRNAs) with a primary role in transposon suppression^16–18^. TE piRNAs associate with MIWI2 and MILI, and guide transcriptional and posttranscriptional transposon silencing, respectively. The second population of piRNAs, termed pachytene piRNAs, comprises the vast majority of piRNAs in adult mouse testes^2–5^. These piRNAs accumulate rapidly at the pachytene stage of meiosis^19^. Unlike TE piRNAs, pachytene piRNAs are transposon sequence-poor and associate with MILI and MIWI. Although a definite function in transposon regulation has not been established^20^, emerging evidence indicates pachytene piRNAs may promote spermatogenesis by regulating mRNAs and long non-coding RNAs in mouse testes^8–11^. The precise biological function of pachytene piRNAs is still not well-understood^21^.

Pachytene piRNAs comprise the largest and most diverse population of small non-coding RNAs in the testis with more than two million distinct piRNA species^19^. These piRNAs are primarily generated from hundreds of unique genomic loci (piRNA clusters) through a primary processing pathway^3,19,22^, and transcription factor A-MYB plays a critical role in driving the transcription of the bulk of pachytene piRNA precursors^19^. Notably, pachytene piRNAs have thus far only been identified in mammals, but not in well-studied flies and worms. Despite this, accumulating evidence indicates that conserved piRNA biogenesis factors are expressed and active during the pachytene stage of meiosis and may contribute to pachytene piRNA biogenesis^20,23–27^. Additionally, the inventory of piRNA biogenesis factors during this period is still not complete^28,29^.

Tudor domain proteins play conserved roles in regulating the piRNA pathway and spermatogenesis by interacting with the PIWI proteins^12,30^. Tudor domain proteins bind methylated arginines on PIWI proteins through the Tudor domain and promote the formation and localization of piRNA processing complex. The essential function of Tudor domain proteins has been implicated in distinct steps of piRNA biogenesis. Among them, TDRKH is required for pre-piRNA trimming^25^. RNF17 suppresses piRNA ping-pong mechanism in meiotic cells^24^. TDRD1, TDRD9, and TDRD12 are involved in ping-pong amplification and secondary piRNA production during embryonic/perinatal piRNA biogenesis^31–33^.

TDRD5 is a Tudor domain protein implicated in spermatogenesis and male fertility^34,35^. TDRD5 null mutations impair transposon silencing and disrupt spermiogenesis^35^. However, the role of TDRD5 in piRNA biogenesis has not been established. By global deletion of *Tdrd5* in mice and conditional inactivation of *Tdrd5* in postnatal germ cells, we discovered a critical role for TDRD5 in piRNA biogenesis. TDRD5 directly binds piRNA precursors and is required for the production of the bulk of pachytene piRNAs during meiosis. TDRD5 exerts it role by selectively controlling the processing of a large subset of the most abundantly expressed pachytene piRNA precursors. We also provide evidence that pachytene piRNA precursor processing contains two genetically separable steps: 5′ end processing and the processing of the rest of the piRNA precursors. These observations reveal previously unknown mechanistic features of pachytene piRNA biogenesis supporting spermatogenesis and male fertility.

## Results

### Reduced piRNA production in *Tdrd5* null mice

Because *Tdrd5* is regulated by the master piRNA transcription factor A-MYB during meiosis^19^, we speculated that TDRD5 is involved in piRNA biogenesis. To test this hypothesis, we generated *Tdrd5* null mice (*Tdrd5*^*KO*^) using an embryonic stem cell line with targeted *Tdrd5* mutation (Supplementary Fig. 1a). Western blot analysis confirmed the absence of TDRD5 proteins in *Tdrd5*^*KO*^ mice (Supplementary Fig. 1b). As observed before^35^, TDRD5 deficiency caused spermatogenic arrest at either zygotene (severe phenotype) or round spermatid (mild phenotype) stages of spermatogenesis (Supplementary Fig. 1c and d). We next examined the total piRNA by gel electrophoresis. Testes from adult *Tdrd5*^*KO*^ mice with severe phenotype lacked piRNA-producing cells (pachytene spermatocytes and round spermatids) and associated piRNA production (Supplementary Fig. 1e, right). Testes from adult *Tdrd5*^*KO*^ mice with mild phenotypes contained pachytene spermatocytes and round spermatids, but total piRNA levels were significantly reduced as compared to the wild type (Supplementary Fig. 1e, left). This suggests that TDRD5 participates in adult piRNA biogenesis.

### Loss of TDRD5 in postnatal germline impairs spermiogeneis

**Fig. 1:**
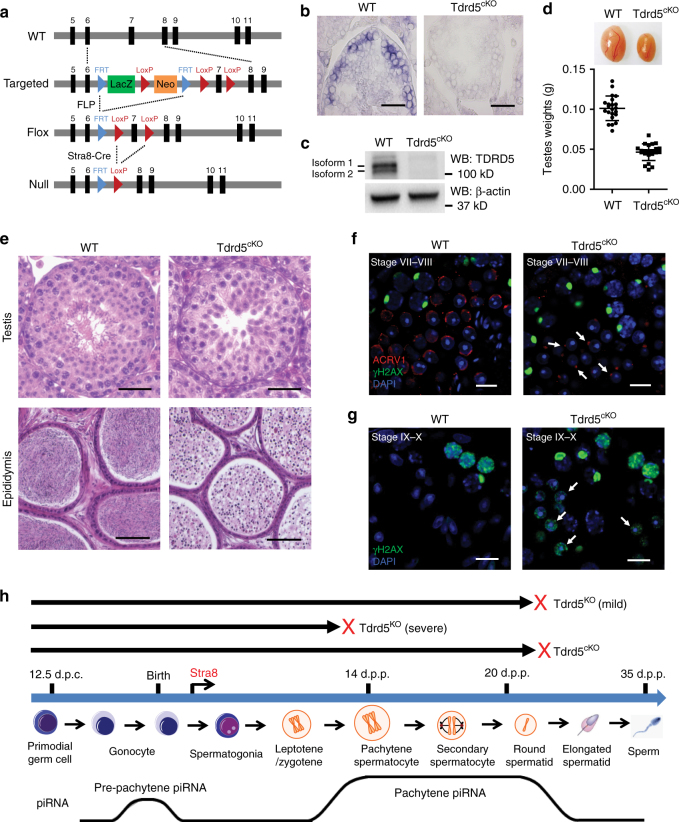
Conditional inactivation of *Tdrd5* in postnatal male germ cells leads to spermatogenic arrest and male infertility in mice. **a** A schematic diagram showing the targeting strategy for the generation of a *Tdrd5* conditional allele. Cre-mediated deletion removed the exon 7 of *Tdrd5* and generated a protein null allele. **b** In situ hybridization of Tdrd5 mRNA in adult wild-type (WT) and *Tdrd5*^*cKO*^ testes. Scale bar, 40 μm. **c** Western blotting of TDRD5 expression in adult WT and *Tdrd5*^*cKO*^ testes. β-actin served as a loading control. **d** Testicular atrophy in *Tdrd5*^*cKO*^ mice. Testis sizes and weights of adult WT and *Tdrd5*^*cKO*^ mice are shown. *n* = 22. Error bars represent s.e.m. **e** Spermatogenic arrest at the round spermatid stage in *Tdrd5*^*cKO*^ testes. Hematoxylin and eosin stained testis and epididymis sections from adult WT and *Tdrd5*^*cKO*^ mice are shown. Scale bars, 40 μm (top) and 100 μm (bottom). **f** Spermatogenic arrest at the round spermatid stage in *Tdrd5*^*cKO*^ testes. Co-immunostaining of ACRV1 and γH2AX in stage VII–VIII seminiferous tubule from WT and *Tdrd5*^*cKO*^ testes. DNA was stained by DAPI. Scale bar, 10 μm. **g** Loss of TDRD5 causes DNA damage in arrested round spermatids. Immunostaining of γH2AX in stage IX–X seminiferous tubule from WT and *Tdrd5*^*cKO*^ testes. DNA was stained by DAPI. Scale bar, 10 μm. **h** The timeline of mouse spermatogenesis with red crosses representing the arrested spermatogenic stages in *Tdrd5*^*KO*^ and *Tdrd5*^*cKO*^ testes

TDRD5 is expressed in both embryonic and meiotic male germ cells^34,35^. TDRD5 null mutation affected piRNA production and spermatogenesis at both stages, complicating the conclusion for a clear effect of TDRD5 on piRNA biogenesis. To test for a direct role of TDRD5 in pachytene piRNA biogenesis, we generated *Tdrd5* conditional knockout mice in which *Tdrd5* becomes deleted in postnatal day 3 male germ cells by *Stra8*-Cre (Fig. 1a). We generated mice with a *Tdrd5* knockout-first allele (*Tdrd5*^tm1a^) and mice with a *Tdrd5* conditional allele (*Tdrd5*^*fl*^) via FLP recombination (Fig. 1a). In the *Tdrd5*^*fl*^ allele, exon 7 of *Tdrd5* is flanked by two loxP sites, by combining with *Stra8*-Cre, we obtained *Stra8*-Cre^+^, *Tdrd5*^*fl/*−^ conditional knockout mice (refer to as *Tdrd5*^*cKO*^) in which TDRD5 is deleted in all adult male germ cell lineages (Fig. 1a, Supplementary Fig. 2). Successful inactivation of TDRD5 in *Tdrd5*^*cKO*^ mice was confirmed by in situ hybridization and western blotting, which revealed the absence of both TDRD5 mRNA and protein in adult *Tdrd5*^*cKO*^ testes (Fig. 1b and c). *Tdrd5*^*cKO*^ male mice exhibited atrophied testes with an average of 50% of wild-type control testis weight (Fig. 1d), and were infertile due to germ cell arrest at the round spermatid stage (Fig. 1e). No elongating spermatids or spermatozoa were formed in *Tdrd5*^*cKO*^ seminiferous epithelium (Fig. 1e). As a result, only round spermatid-like cells could be observed in *Tdrd5*^*cKO*^ epididymides (Fig. 1e). In *Tdrd5*^*cKO*^ testes, round spermatids arrested before step 5 as proacrosome granules but not acrosome caps were observed in arrested spermatids (Fig. 1f). Subsequently, arrested *Tdrd5*^*cKO*^ round spermatids showed pronounced DNA damage after reaching seminiferous epithelium stage VIII (Fig. 1g). These results indicate that postnatal expression of TDRD5 is essential for spermiogenesis (Fig. 1h).

### TDRD5 is essential for pachytene piRNA biogenesis

**Fig. 2:**
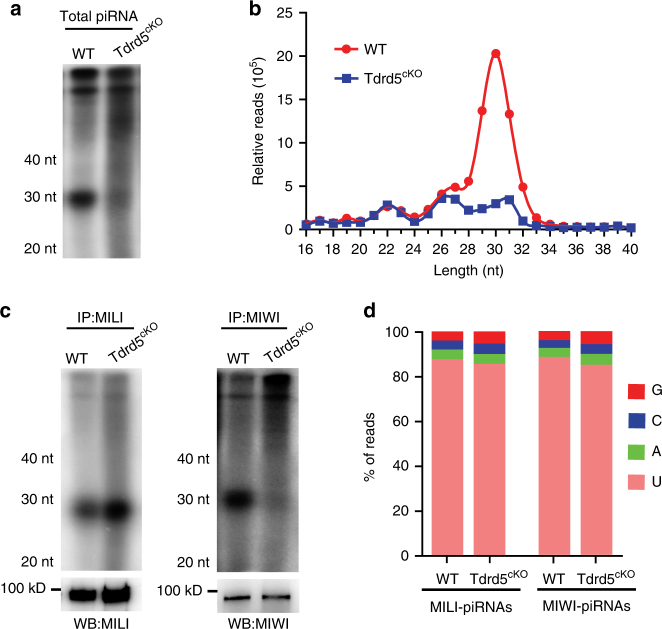
Postnatal male germ cell-expressed TDRD5 is essential for pachytene piRNA biogenesis. **a** Total RNA from adult WT and *Tdrd5*^*cKO*^ testes was end-labeled with [^32^P]-ATP and detected by 15% TBE urea gel and autoradiography. nt, nucleotide. **b** Size distribution of small RNA libraries from adult WT and *Tdrd5*^*cKO*^ testes. Data were normalized by microRNA reads (21–23 nt). **c** MILI- and MIWI-bound piRNAs from adult WT and *Tdrd5*^*cKO*^ testes. Small RNAs were isolated from immunoprecipitated MILI and MIWI RNPs and were end-labeled with [^32^P]-ATP and detected by 15% TBE urea gel and autoradiography. Western blotting was performed with MILI and MIWI antibodies to show immunoprecipitation efficiency. **d** Nucleotide composition of first nucleotide of MILI-piRNAs and MIWI-piRNAs in adult WT and *Tdrd5*^*cKO*^ testes. The piRNAs in *Tdrd5*^*cKO*^ exhibited a 5′ end U bias at position 1

We next examined the effect of postnatal germ cell-specific TDRD5 loss on piRNA biogenesis. Radiolabeling of total RNA isolated from adult wild-type and *Tdrd5*^*cKO*^ testes revealed that the total piRNA production was severely reduced in *Tdrd5*^*cKO*^ testes (Fig. 2a). However, the piRNAs produced in *Tdrd5*^*cKO*^ testes appeared to be of normal size distribution (Fig. 2a). Sequencing of small RNAs from wild-type and *Tdrd5*^*cKO*^ total RNA revealed that two predominate populations comprised the remaining piRNAs in *Tdrd5*^*cKO*^ testes, corresponding to 25–28 nt MILI-bound piRNAs (MILI-piRNAs) and 29–32 nt MIWI-bound piRNAs (MIWI-piRNAs) (Fig. 2b). To quantify the relative abundance of total piRNA in wild-type and *Tdrd5*^*cKO*^ testes, we used total microRNAs (miRNAs), a widely used reference small RNA population^36^, to normalize piRNAs in each library. After normalization to the total number of miRNA reads, the *Tdrd5*^*cKO*^ piRNA population was ~25–30% of wild-type level. The expression of the 25–28 nt piRNAs was unaffected while the 29–32 nt piRNAs was reduced in *Tdrd5*^*cKO*^. This pattern was confirmed when we examined radio-labeled piRNAs bound to MILI or MIWI (Fig. 2c). MILI expression and localization were unaffected in *Tdrd5*^*cKO*^ testes (Supplementary Fig. 3a and c). MIWI expression was decreased with largely unaffected localization pattern in *Tdrd5*^*cKO*^ testes (Supplementary Fig. 3b and c). The pattern of piRNA deficiency in *Tdrd5*^*cKO*^ testes was similar to that of *Miwi* knockout (*Miwi*^*KO*^) mice, which exhibit normal MILI-piRNAs but devoid of MIWI-piRNAs^37,38^ (Supplementary Fig. 4). The piRNA defect in *Tdrd5*^*cKO*^ mice differs from the effect seen in pachytene piRNA biogenesis factor *Mov10l1*^*cKO*^ mice, which yields complete loss of both MILI-piRNAs and MIWI-piRNAs^20^. RNA-seq of immunoprecipitated MILI-piRNAs and MIWI-piRNAs from *Tdrd5*^*cKO*^ testes revealed a 5′ U-bias, a piRNA 5′ end signature observed in wild-type testes (Fig. 2d). This indicates that *Tdrd5*^*cKO*^ piRNA 5′ formation is normal. Size distribution of *Tdrd5*^*cKO*^ MILI-piRNAs and MIWI-piRNAs was comparable to that of the wild type, suggesting piRNA trimming was not significantly affected by TDRD5 deficiency (Supplementary Fig. 5). Together, these data establish a critical role for TDRD5 in pachytene piRNA biogenesis.

### TDRD5 deficiency selectively reduces cluster-derived piRNAs

**Fig. 3:**
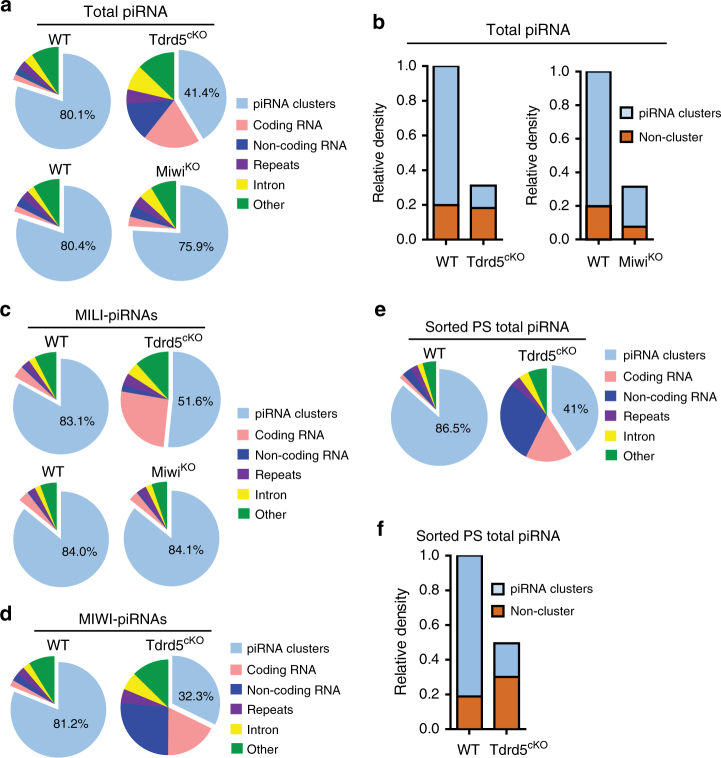
TDRD5 selectively controls the production of piRNA cluster-derived pachytene piRNAs. **a** Genomic annotation of total piRNA from adult WT, *Tdrd5*^*cKO*^, and *Miwi*^*KO*^ testes. piRNA clusters: 214 piRNA clusters defined by Li et al.^19^. **b** Relative abundance of total piRNA from adult WT, *Tdrd5*^*cKO*^, and *Miwi*^*KO*^ testes normalized by miRNA. Note specific reduction of piRNA cluster-derived piRNAs in *Tdrd5*^*cKO*^ testes. **c** Genomic annotation of MILI-bound piRNAs from adult WT, *Tdrd5*^*cKO*^, and *Miwi*^*KO*^ testes. **d** Genomic annotation of MIWI-bound piRNAs from adult WT and *Tdrd5*^*cKO*^ testes. **e** Genomic annotation of total piRNA from sorted pachytene spermatocytes (PS) from WT and *Tdrd5*^*cKO*^ testes. **f** Relative abundance of total piRNA from sorted pachytene spermatocytes from WT and *Tdrd5*^*cKO*^ testes normalized by miRNA

We further characterized the piRNAs produced in *Tdrd5*^*cKO*^ mice by mapping the reads to the mouse genome. In adult wild-type testes, 80% piRNAs are derived from recently defined 214 piRNA clusters as seen previously^19^ (Fig. 3a, Supplementary Table 1). But there was a specific reduction in the percentage of 214 piRNA cluster-derived piRNAs in *Tdrd5*^*cKO*^ testes (Fig. 3a). This contrasts with an increase in piRNA percentage from other piRNA sources including coding RNAs, non-coding RNAs, repeats, and introns. To confirm that the reduction in piRNA cluster-derived piRNAs was specific for TDRD5 deficiency, we examined the piRNA composition in *Miwi*^*KO*^ mice, which exhibit similar germ cell arrest and levels of overall piRNA reduction (Supplementary Fig. 4)^37^. The percentage of cluster-derived piRNAs in *Miwi*^*KO*^ was equivalent to that of wild type (Fig. 3a). This indicates that, unlike MIWI deficiency, TDRD5 deficiency selectively reduces piRNA production from piRNA clusters. When normalized to miRNA expression, the reduction in total piRNA expression was similar between TDRD5 and MIWI deficiency. piRNAs mapping to “non-cluster” regions (coding RNAs, non-coding RNAs, repeats, introns, and other) were not decreased, indicating that piRNA reduction in *Tdrd5*^*cKO*^ mice was specific to piRNA clusters (Fig. 3b). By contrast, piRNAs derived from piRNA clusters and non-cluster regions were both reduced in *Miwi*^*KO*^ testes (Fig. 3b), further indicating the special role of TDRD5 to selectively control piRNA production from piRNA clusters. Analysis of MILI-piRNAs in *Tdrd5*^*cKO*^ testes showed the same specific percentage reduction of cluster-derived piRNAs (Fig. 3c), even when MILI proteins were loaded with the similar amount of piRNAs in *Tdrd5*^*cKO*^ testes (Fig. 2c). This also differs from *Miwi*^*KO*^, in which the percentage of MILI-piRNAs from different origin was not affected (Fig. 3c). The percentage of cluster-derived piRNAs within *Tdrd5*^*cKO*^ MIWI-piRNAs was also reduced (Fig. 3d). We next sorted and purified pachytene spermatocytes to confirm that the selective reduction in piRNA cluster-derived piRNAs occurs in this main pachytene piRNA-producing cell type. After RNA-seq of total small RNAs from wild-type and *Tdrd5*^*cKO*^ spermatocytes, we analyzed piRNA length distribution and composition (Supplementary Fig. 6). Similar with the results from whole testes, *Tdrd5*^*cKO*^ spermatocytes displayed a normal amount of 25–28 nt small RNAs corresponding to MILI-piRNAs, and much lower amount of 29–32 nt small RNAs corresponding to MIWI-piRNAs as compared to the wild type (Supplementary Fig. 6b). After mapping these total piRNA reads to the mouse genome, *Tdrd5*^*cKO*^ spermatocytes displayed a specific decrease in cluster-derived piRNAs normalized by miRNAs (Fig. 3e, f). Collectively, these results indicate that TDRD5 is a key pachytene piRNA biogenesis factor required specifically for the production of piRNA cluster-derived, but not other source-derived piRNAs during the pachytene stage of male meiosis.

### TDRD5 selectively regulates top piRNA-producing clusters

**Fig. 4:**
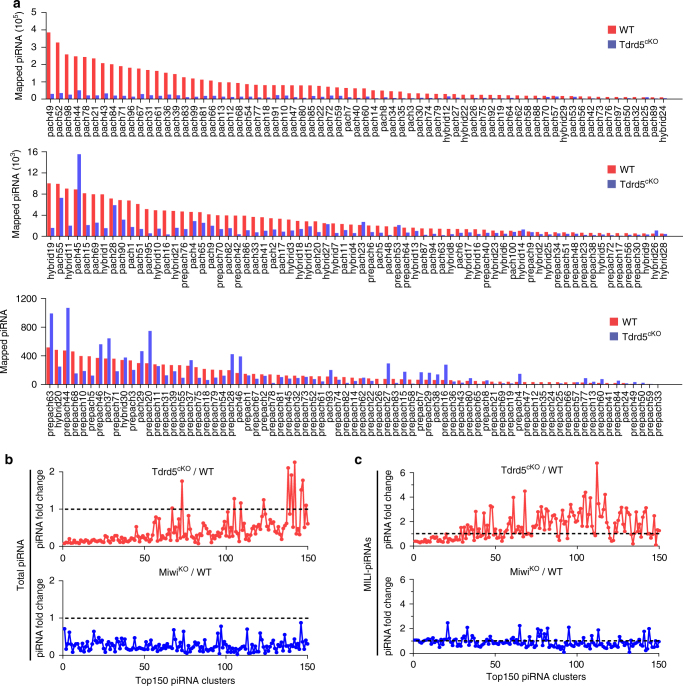
Loss of TDRD5 selectively reduces piRNA production from a subset of top-piRNA-producing loci within 214 piRNA clusters. **a** Shown is the number of piRNA reads mapped to 214 individual piRNA clusters from small RNA libraries of WT and *Tdrd5*^*cKO*^ testes. piRNA reads were normalized by the miRNA counts of each small RNA library. piRNA clusters are ranked by the their piRNA abundances in WT library. **b** Comparison of piRNA fold change (*Tdrd5*^*cKO*^*/*WT or *Miwi*^*KO*^/WT) of top150 piRNA-producing clusters from indicated total small RNA libraries. piRNA reads were normalized by miRNA counts of each library. *X*-axis represents the top150 piRNA clusters ranked by their piRNA abundances in WT library in descending order. **c** Comparison of piRNA fold change (*Tdrd5*^*cKO*^*/*WT or *Miwi*^*KO*^/WT) of top150 piRNA-producing clusters from indicated MILI-piRNA libraries. piRNA reads were normalized by total reads of each library. *X*-axis represents the top150 piRNA clusters ranked by their piRNA abundances in WT library in descending order

To assess the effect of TDRD5 loss on piRNA production from individual piRNA clusters, we analyzed piRNA reads from wild-type and *Tdrd5*^*cKO*^ small RNA libraries mapped to each of the 214 piRNA clusters^19^. After normalization by miRNA counts of each library, we directly compared the number of wild-type and *Tdrd5*^*cKO*^ piRNA reads mapped to each piRNA cluster (Fig. 4a). In wild-type testes, top 50 piRNA-producing clusters give rise to vast majority (>90%) of 214 cluster-derived piRNAs (Fig. 4a). In *Tdrd5*^*cKO*^ testes, piRNAs produced from these top 50 piRNA-producing clusters were uniformly reduced by an average of sevenfold. In contrast, the piRNAs mapped to low-piRNA-producing clusters were less affected by TDRD5 deficiency, particularly the 84 prepachytene and 30 hybrid piRNA clusters previously defined within these 214 piRNA clusters^19^ (Fig. 4a, b). This indicates that loss of TDRD5 selectively affects piRNA production from a subset of most abundantly expressed pachytene piRNA precursors. To confirm this selective effect on piRNA reduction is unique to TDRD5, we examined the pattern of piRNA reduction in individual piRNA clusters in *Miwi*^*KO*^ testes. Unlike the effect of TDRD5 deficiency on total piRNA production, Miwi deficiency had a uniform reduction effect on both the high-piRNA-producing clusters and the low-piRNA-producing clusters (Fig. 4b). Analysis of the MILI-piRNAs from *Tdrd5*^*cKO*^ testes and *Miwi*^*KO*^ testes revealed a similar trend. TDRD5 deficiency caused selective reduction of MILI-piRNA production from high-piRNA-producing clusters while MIWI deficiency did not globally affect MILI-piRNAs from almost all clusters (Fig. 4c). These data together indicate that, within the 214 piRNA clusters, TDRD5 deficiency selectively ablates piRNA production from a large subset of most highly expressed piRNA clusters, which correlates with A-MYB transcriptionally controlled piRNA clusters.

### Genetically separable steps in pachytene piRNA processing

**Fig. 5:**
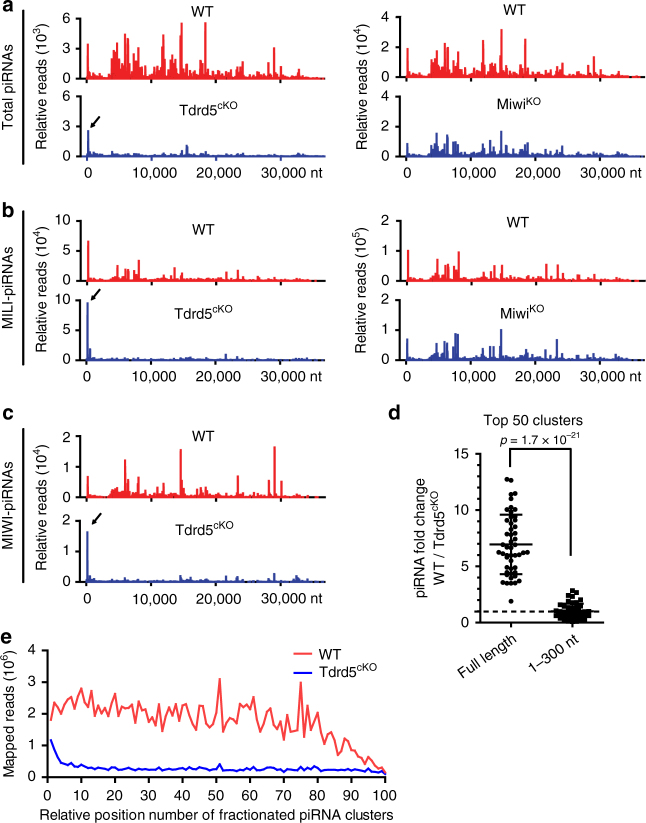
TDRD5 deficiency causes differential piRNA biogenesis within single-piRNA clusters. **a** Distribution of piRNA reads mapping to a representative piRNA cluster (cluster 43) from adult WT, *Tdrd5*^*cKO*^, and *Miwi*^*KO*^ mice. The data were normalized by miRNA counts of each small RNA library pair. Arrows indicate the successful production of piRNAs from 5′ end but not across the entire length of the precursor transcript in *Tdrd5*^*cKO*^ testes. **b** Distribution of MILI-piRNAs mapped to a representative piRNA cluster (cluster 43) from adult WT, *Tdrd5*^*cKO*^, and *Miwi*^*KO*^ mice. The data were normalized by total small RNA reads from each library pair. **c** Distribution of MIWI-piRNAs mapped to a representative piRNA cluster (cluster 43) from adult WT and *Tdrd5*^*cKO*^ mice. The data were normalized by total small RNA reads from each library pair. **d** Comparison of piRNA fold change (WT/*Tdrd5*^*cKO*^) from 5′ 300 nt vs full-length transcripts from top50 piRNA-producing clusters. The data were normalized by miRNA counts of WT and *Tdrd5*^*cKO*^ small RNA libraries. The *p*-value was calculated using paired *t*-test. Error bars represent s.e.m. **e** Total piRNA reads from WT and *Tdrd5*^*cKO*^ libraries were mapped to 214 piRNA clusters. The density plots of mapped piRNA reads at relative positions of 214 piRNA clusters are shown. The data were normalized by miRNA counts of each small RNA library pair

To investigate whether TDRD5 deficiency affects piRNA production uniformly within single-piRNA clusters, we analyzed *Tdrd5*^*cKO*^ piRNA densities across the lengths of representative high-piRNA-producing clusters. As an example, the pach43 cluster (also named 17-qA3.3-27363.1) is one of the most abundantly expressed piRNA cluster. Unexpectedly, although the total amount of piRNAs produced was significantly reduced from this cluster, the 5′ ends of precursor RNAs (within ~300 bp of transcription start site) could still produce piRNAs at levels comparable to wild-type controls. The rest of piRNA-producing regions in this precursor generated very little piRNAs (Fig. 5a). Similar results were observed from other representative piRNA clusters (Supplementary Fig. 7a). This is highly surprising and represents a special form of piRNA biogenesis defect that occurs within single clusters. The normal presence of precursor 5′ derived piRNAs and the selective depletion of piRNAs from the remainder of piRNA precursor in *Tdrd5*^*cKO*^ mice is different from the piRNA defect in *Miwi*^*KO*^ mice, which showed proportionally reduced piRNA density across the entire length of piRNA cluster (Fig. 5a, Supplementary Fig. 7b). We next sought to confirm this unique piRNA defect occurred in both MILI- and MIWI-bound piRNAs. Analysis of MILI-piRNAs in *Tdrd5*^*cKO*^ mice showed the same trend as observed in total piRNAs, with piRNA production from 5′ 300 nt regions being almost unchanged, while the piRNAs from the rest length of the precursor had a significant decrease (Fig. 5b, Supplementary Fig. 8). Similar results were also observed for *Tdrd5*^*cKO*^ MIWI-piRNAs (Fig. 5c, Supplementary Fig. 8b). To confirm that differential reduction in piRNA production from a single-piRNA cluster is common for all of the high-piRNA-producing clusters affected by TDRD5 loss, we analyzed the piRNA fold change within the 5′ end 300 nt region or across full-length of the transcript for the top 50 piRNA precursors. Although the total amount of piRNAs produced from whole precursors was reduced to an average of sevenfold, the amount of piRNAs produced from the 5′ ends of precursor RNAs within 300 nt were almost unchanged (Fig. 5d). We further analyzed piRNA densities from all 214 piRNA precursors. We divided each of the 214 piRNA precursor RNAs into 100 fragments of equal length and mapped piRNAs from wild-type and *Tdrd5*^*cKO*^ piRNA libraries to each of the 100 fragments from each piRNA precursor. Results indicate that the 5′ ends of precursor RNAs could still produce piRNAs at levels comparable to wild-type controls, while the rest of piRNA-producing regions in these precursors generated very little piRNAs (Fig. 5e, Supplementary Fig. 9). Together, these data reveal that pachytene piRNA production within a single precursor can be genetically separated into at least two steps: 5′ end processing, and the processing of the rest of the transcript.

### TDRD5 interacts with PIWI proteins

**Fig. 6:**
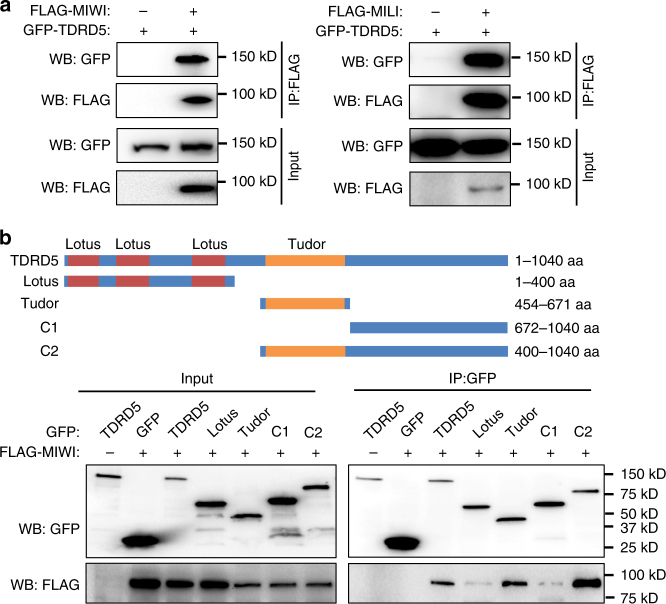
TDRD5 interacts with PIWI proteins. **a** TDRD5 interacts with MIWI and MILI. HEK293T cells were transfected with indicated plasmids. Forty-eight hours after transfection, immunoprecipitation was performed using anti-FLAG resin. GFP-TDRD5 and FLAG tagged proteins were detected by western blotting with anti-GFP and anti-FLAG antibodies. **b** TDRD5 interacts with MIWI through the Tudor domain. HEK293T cell was transfected with indicated plasmids. Forty-eight hours after transfection, immunoprecipitation was performed using anti-GFP resin. GFP-tagged TDRD5 fragments and FLAG-MIWI proteins were detected by western blotting using anti-GFP and anti-FLAG antibodies

We next sought to understand the potential mechanism by which TDRD5 plays its role in the piRNA pathway. The TDRD5 protein contains one Tudor domain and three LOTUS domains^39–43^. The Tudor domain displays conserved binding to PIWI proteins in animal germ cells^30^. Since TDRD5 regulates the production of both MILI-piRNAs and MIWI-piRNAs, we examined its direct association with PIWI proteins. When ectopically co-expressed with MILI or MIWI in HEK293T cells, TDRD5 was detected in both MILI and MIWI immunoprecipitates, indicating its ability to interact with PIWI proteins (Fig. 6a). To test whether the interaction is mediated by the Tudor domain, we used a series of truncated proteins of TDRD5 to examine interactions with MIWI. The Tudor domain, but not other regions of TDRD5, was mainly responsible for interaction with MIWI (Fig. 6b). These data suggest that TDRD5 could enter into the piRNA pathway by interacting with PIWI proteins.

### TDRD5 directly binds piRNA precursors

**Fig. 7:**
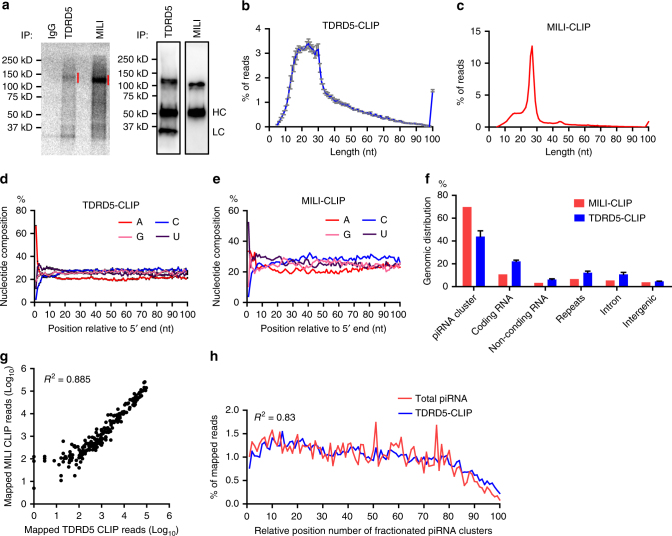
TDRD5 directly binds piRNA precursors. **a** Autoradiography (left) and western blot (right) of TDRD5-RNA and MILI-RNA complexes from CLIPs. IgG served as negative control. Red lines indicate the corresponding RNA regions that were extracted from the membrane. HC, Ig heavy chain; LC, Ig light chain. **b** Size distribution of RNA reads from TDRD5-CLIP libraries. *n* = 3; Error bars represent s.e.m. **c** Size distribution of RNA reads from MILI-CLIP library. **d** Nucleotide composition of TDRD5-CLIP reads. *n* = 3; Error bars represent s.e.m. **e** Nucleotide composition of MILI-CLIP reads. **f** Genomic annotation of TDRD5-CLIP reads and MILI-CLIP reads. TDRD5-CLIP libraries, *n* = 3; Error bars represent s.e.m. **g** Scatter plot of piRNA reads mapped to 214 individual piRNA clusters from TDRD5-CLIP and MILI-CLIP libraries. Pearson correlation (*R*^2^) is shown. **h** Density plots of total piRNA reads and TDRD5-CLIP reads mapped to each of the 100 positions proportionally divided in all 214 piRNA clusters. Each of the 214 piRNA clusters is equally divided into 100 sequence fragments. The total piRNA reads or TDRD5-CLIP reads were mapped to each fragment of each piRNA cluster and total mapped reads for each position were added up for all 214 clusters. Pearson correlation (*R*^2^) is shown

To test the hypothesis that TDRD5 directly participates in piRNA precursor processing, we examined the potential association of piRNA precursors with TDRD5. We performed UV cross-linking immunoprecipitation of TDRD5 in wild-type and *Tdrd5*^*cKO*^ testes and examined the expression levels of several piRNA precursors by RT-PCR. The piRNA precursor RNAs were specifically associated with TDRD5 immunoprecipitates from wild-type testes, but not detected in the IgG control immunoprecipitates from wild-type testes or TDRD5 immunoprecipitates from *Tdrd5*^*cKO*^ testes (Supplementary Fig. 10). These results suggest that TDRD5 could associate with piRNA precursors. To further test whether TDRD5 directly binds piRNA precursors, we performed high-throughput sequencing of RNA isolated by cross-linking immunoprecipitation (HITS-CLIP or CLIP-seq) in testes from adult wild-type mice^44^ (Fig. 7, Supplementary Fig. 11). We first detected TDRD5-specific protein-RNA complexes by CLIP and autoradiography (Fig. 7a). MILI-CLIP was used as a control to represent a known piRNA pathway RNA-binding protein that directly binds RNA (Fig. 7a). CLIP results indicate that, like MILI, TDRD5 directly binds RNA. We next constructed CLIP-seq libraries using RNA isolated from TDRD5-CLIP and MILI-CLIP complexes and performed deep sequencing. Length distribution of TDRD5 CLIP reads displayed a broader length range as compared to MILI-CLIP reads, which primarily contained mature piRNAs of 25–28 nt in length (Fig. 7b, c). TDRD5-CLIP reads contained a predominate A at the first nucleotide position (Fig. 7d), indicative of a signature of digestion by endogenous testicular nucleases^21,44^. In contrast, MILI-CLIP reads showed an expected strong preference for U as the first nucleotide, a signature of mature piRNAs (Fig. 7e). We further analyzed the genomic origin of TDRD5-CLIP and MILI-CLIP reads (Fig. 7f). Over 40% of TDRD5-CLIP reads were mapped to 214 piRNA clusters, consistent with its critical role in piRNA precursor processing. Within the 214 piRNA clusters, the mapped TDRD5-CLIP and MILI-CLIP reads displayed a linear correlation with the amount of piRNA production from each cluster (Fig. 7g). To explore whether TDRD5 has a preference in binding to certain regions within individual piRNA precursor RNAs, we analyzed the densities of TDRD5-CLIP and MILI-CLIP reads across the lengths of representative high-piRNA-producing clusters. The TDRD5-CLIP read densities at each position were highly correlated with MILI-CLIP read densities, indicating that TDRD5 binding sites cover the majority of piRNA-producing sites on piRNA precursors (Supplementary Fig. 12). We further analyzed the densities of TDRD5-CLIP reads and mature piRNA reads from all 214 piRNA precursors. We divided each of the 214 piRNA precursor RNAs into 100 fragments of equal length and mapped TDRD5-CLIP reads and mature piRNA reads to each of the 100 fragments from each piRNA precursor. The densities of mature piRNA reads correlated significantly (*R*^2^ = 0.83) with that of TDRD5-CLIP reads throughout the length of all 214 piRNA precursors, indicating that the binding of TDRD5 to piRNA precursors was functionally coupled with their processing into piRNAs (Fig. 7h). Interestingly, these results also reveal that TDRD5 could bind to any region within the precursor, including ~300 bp of transcription start site, lacking a clear preference in binding to any specific regions in each precursor (Supplementary Fig. 12, Fig. 7h). Together, these data indicate that TDRD5 is an RNA-binding protein that associates with piRNA precursors along their entire lengths. Thus, TDRD5 could regulate piRNA biogenesis through direct association with piRNA precursors.

### piRNA precursors are not accumulated in *Tdrd5*^*cKO*^ testes

**Fig. 8:**
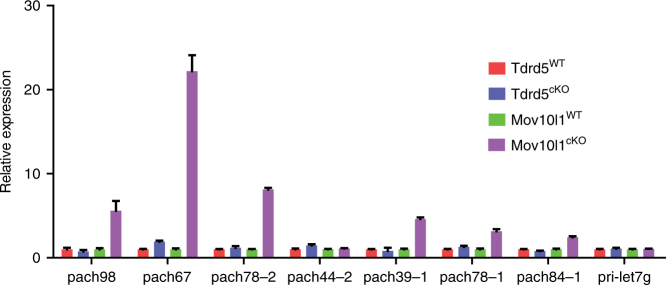
Accumulation of piRNA precursors in *Mov10l1*^*cKO*^ but not in *Tdrd5*^*cKO*^ testes. Total RNA was isolated from testes of indicated animals. Quantitative RT-PCR was performed to detect indicated piRNA precursors. miRNA precursor pri-let7g served as a control. *n* = 3; error bars represent s.e.m.

MOV10L1, an RNA helicase that binds and unwinds piRNA precursors, is required for the production of the entire population of pachytene piRNAs in mice^20,44^. Conditional ablation of MOV10L1 in postnatal male germ cells causes a complete blockade of piRNA precursor processing, resulting in loss of mature piRNAs and corresponding piRNA precursor accumulation^20,44^. To investigate whether loss of TDRD5 has any effect on piRNA precursor abundance, we analyzed piRNA precursor levels in wild-type and *Tdrd5*^*cKO*^ testes (Fig. 8). piRNA precursor expression levels in *Tdrd5*^*cKO*^ testes were not different from the wild type. This contrasts with the significantly elevated piRNA precursor levels in *Mov10l1*^*cKO*^ testes (Fig. 8). The difference in piRNA precursor accumulation suggests distinct roles for TDRD5 and MOV10L1 in pachytene piRNA precursor processing. It is likely that loss of TDRD5 results in the dissociation of piRNA precursors from the piRNA processing complex, which leads to subsequent degradation.

## Discussion

Our data reveal an essential role for Tudor domain protein TDRD5 in piRNA biogenesis in mice. Rather than controlling the production of the whole piRNA population, TDRD5 selectively regulates the piRNA production from a subset of the most abundant piRNA clusters during meiosis. We also discovered that TDRD5 deficiency genetically uncouples pachytene piRNA precursor 5′ end processing from the remainder the precursor. These two discoveries place TDRD5 as a unique protein among known piRNA biogenesis factors and provide insight into the mechanism of mammalian pachytene piRNA biogenesis.

After transcription, pachytene piRNA precursors are believed to be transported from the nucleus to enter intermitochondrial cement (IMC) in cytoplasm for processing. Our results reveal a function of TDRD5 downstream of piRNA precursor recruitment to IMC and upstream of piRNA loading, trimming and maturation. This is supported by the observation that there was relative normal production of precursor 5′ end-derived piRNAs from TDRD5-regulated single piRNA precursors, and that late steps of piRNA biogenesis were not apparently impaired in piRNAs produced in *Tdrd5*^*cKO*^ testes. This places the function of TDRD5 at the start of piRNA processing after precursor entry into piRNA processing complex (PPC). Given the localization of TDRD5 at IMC^35^ and its interaction with PIWI proteins, we propose here TDRD5 is a critical component of the pachytene PPC that regulates a large subset of the most abundantly expressed piRNA precursors funneled through IMC (Supplementary Fig. 13). We propose that the entire pachytene piRNA precursors are classified into TDRD5-regulated and TDRD5-independent sub-populations that are differentially processed. TDRD5-regulated piRNA precursors comprise most of the top piRNA-producing precursors transcribed from intergenic piRNA clusters. Regulated by TDRD5, these precursors account for the most abundant piRNA species produced in wild-type testes. Other piRNA precursors emanating from other low-piRNA producing loci are processed independently of TDRD5, accounting for their insensitivity to TDRD5 deficiency. For TDRD5-regulated precursors, despite of the failure in processing and drastic reduction in mature piRNAs, we did not observe corresponding piRNA precursor accumulation. This contrasts with MOV10L1 deficiency, in which unprocessed piRNA precursors are abundantly accumulated^20^. It is likely that unprocessed piRNA precursors in *Tdrd5*^*cKO*^ testes are degraded, which suggests a role for TDRD5 in piRNA precursor stabilization. This is consistent with our CLIP-seq data demonstrating the direct association of TDRD5 with piRNA precursors. Diminished piRNA production may account for the observed defects in IMC and chromatoid body in TDRD5 deficient germ cells^35^.

Interestingly, despite the severe reduction in piRNA production, individual top piRNA-producing clusters in *Tdrd5*^*cKO*^ still generated significant amount of piRNAs, and the ranking of piRNA amounts produced by each cluster is essentially not changed. This indicates that TDRD5 loss could not reverse the existing advantages that the top piRNA-producing precursors have for being selected and processed by the PPC. This in turn suggests that other unknown protein factor(s) are responsible for the initial selection of piRNA precursors to enter the PPC. We envision that TDRD5 provides an essential layer of selection after piRNA precursors enter the PPC, that is, to further retain/stabilize and facilitate top piRNA-producing precursors for highly efficient processing through direct TDRD5-piRNA precursor interactions.

An important aspect of piRNA biogenesis discovered in this study is that, within a single TDRD5-dependent piRNA cluster, the apparently normal production of piRNAs mapping to the very 5′ end of the piRNA cluster and the diminished production of piRNAs mapping to the rest regions of the cluster. This indicates that the pachytene piRNA precursor processing is genetically separable. The uncoupling of precursor processing within individual clusters was observed in both MILI-piRNAs and MIWI-piRNAs. Why the processing of precursor 5′ end does not require TDRD5 is not known, but it is clear that piRNA precursor recruitment to the PPC continues in the absence of TDRD5. Our TDRD5 CLIP-seq results indicate that TDRD5 directly binds piRNA precursors evenly across their entire lengths with no preferential recognition of specific hot spots, and thereby stabilizes precursors for processing by the PPC critical enzymes MOV10L1 and MitoPLD. Conceivably, the loss of TDRD5 could destabilize precursor retention at the PPC, thereby leading to RNA loss and eventual degradation after precursor 5′ processing. Although we cannot rule out that TDRD5 loss may also affect the processivity of the cleavage enzyme MitoPLD, the direct associate of TDRD5 with piRNA precursors and the absence of piRNA precursor accumulation upon TDRD5 loss are most consistent with a direct role for TDRD5 in piRNA precursor retention/stabilization. Thus, we propose TDRD5 as a core component of the PPC that functions downstream piRNA precursor recruitment to stabilize and enhance precursor processing during piRNA biogenesis.

## Methods

### Ethics statement

All the animal procedures were approved by the Institutional Animal Care and Use Committee of Michigan State University. All experiments with mice were conducted ethically according to the Guide for the Care and Use of Laboratory Animals and institutional guidelines.

### Mouse strains

A *Tdrd5* gene targeted embryonic stem (ES) cell clone, *Tdrd5*^*tm1a*^, was acquired from European Mouse Mutant Cell Repository. *Tdrd5*^*tm1a*^ is a knockout-first allele, which allows the subsequent generation of a conditional *Tdrd5* flox (*Tdrd5*^*fl*^) allele with exon 7 flanked by loxP sites (Supplementary Fig. 1a). To generate *Tdrd5*^*tm1a*^ chimeric mice, ES cells were expanded and injected into C57BL/6 J blastocysts. Chimeric males were bred with C57BL/6 J females to generate heterozygous *Tdrd5*^*tm1a*^ animals.

To generate *Tdrd5*^*fl*^ allele, heterozygous *Tdrd5*^*tm1a*^ animals were bred with FLP-expressing transgenic mice (Jackson laboratory) to remove FRT flanked sequences (Fig. 1a). *Tdrd5*^*fl/+*^ males were bred with *Tdrd5*^*fl/+*^ females to generate homozygous *Tdrd5*^*fl/fl*^ mice. To generate Stra8-Cre *Tdrd5* conditional knockout mice, Stra8-Cre transgenic mice (Jackson Laboratory) were bred with *Tdrd5*^*fl/fl*^ mice using scheme described in Supplementary Fig. 2a. Primers for *Tdrd5*^*fl/fl*^ mice genotyping PCR are: F1: 5′-AGGCTCTAATATGTACCGTCTGAGGG-3′ and R1: 5′-CTATTTCACCATCAACCAATCTAGCC-3′. Wild-type allele produced a 504 bp product; *Tdrd5*^*fl*^ allele generated a 594 bp product. Primers for Stra8-cre PCR (236 bp) were: 5′-GTGCAAGCTGAACAACAGGA-3′ and 5′-AGGGACACAGCATTGGAGTC-3′. A set of primers was used as the internal control (324 bp) in Stra8-cre genotyping PCR: 5′-CTAGGCCACAGAATTGAAAGATCT-3′ and 5′-GTAGGTGGAAATTCTAGCATCATCC-3′. (Supplementary Fig. 2b)

*Miwi* knockout mice, generated in the laboratory of Dr. Haifan Lin^37^, were purchased from Mutant Mouse Resource Research Centers. *Mov10l1* flox (*Mov10l1*^*fl/fl*^) mice, generated in the laboratory of Dr. Eric Olson, were purchased from Jackson Laboratory^45^. To generate *Mov10l1* conditional knockout mice, *Mov10l1*^*fl/fl*^ mice were bred with Stra8-Cre mice (Jackson Laboratory).

### TDRD5 antibody generation

Complimentary DNA corresponding to TDRD5 283–371 aa was cloned into pET-28a (His-tag) and pGEX-4t-1 (GST-tag) vectors. His-tagged recombinant protein was used as an antigen to generate rabbit anti-TDRD5 polyclonal antisera (Pacific Immunology). Antisera were affinity purified with GST-tagged antigen immobilized on beaded agarose using AminoLink Plus immobilization kit (Thermo Scientific).

### Histology

Testes and epididymides from adult wild-type and mutant mice were fixed in Bouin’s fixative and embedded in paraffin. For the histological analysis, sections of 5 μm were cut and stained with hematoxylin and eosin after dewaxing and rehydration.

### Immunofluorescence

Testes were fixed in 4% PFA in PBS overnight at 4 °C and embedded in paraffin. For immunostaining, tissue sections of 5 μm were cut, dewaxed and rehydrated. Antigen retrieval was performed by microwaving the sections in 0.01 M sodium citrate buffer (pH 6.0) for 4 min. Tissue sections were blocked in 5% normal goat serum (NGS) for 30 min after rinsing with PBS. Testis sections were then incubated with primary antibodies diluted in 5% NGS at 37 °C for 2 h. Antibodies used were: rabbit anti-MIWI (1:100; 2079, Cell Signaling Technology), rabbit anti-MILI (1:100; PM044, MBL), rabbit anti-ACRV1 (1:50; 14040-1-AP, Protein Tech) or FITC-conjugated mouse anti-γH2AX (1:500; 16–202 A, Millipore). After washing with PBS, sections were incubated with Alexa Fluor 555 goat anti-rabbit IgG (1:500; A21429, Life Technologies) for 1 h and mounted using Vectorshield mounting media with DAPI (H1200, Vector Laboratories). Confocal fluorescence microscopy was conducted using FluoView 1000 microscope (Olympus, Japan).

### In situ hybridization

Testes were fixed in 4% PFA in PBS overnight at 4 °C, immersed in 30% sucrose, and embedded in O.C.T compound. Sections of 7 μm were cut. Sense and antisense DIG labeled RNA probes were transcribed from a linearized plasmid containing a *Tdrd5* cDNA fragment (nucleotides 2160–2676, GenBank NM_001134741.1) using DIG RNA Labeling Mix (Roche). The probes were denatured for 10 min in hybridization cocktail solution (Amresco) and added to the sections for incubation at 65 °C overnight. Sections were then washed, blocked, and incubated with alkaline phosphatase-conjugated goat anti-DIG Fab fragments (Roche) at 4 °C overnight. The positive signal was visualized by BM Purple (Roche).

### Co-immunoprecipitation

Miwi and Mili/Piwil2 cDNAs were cloned into a modified pcDNA3 vector encoding a FLAG-tag^26^. Full-length Tdrd5 cDNA and partial TDRD5 cDNA fragments were cloned into the pEGFP-C1 vector. 293T cells were transfected with indicated plasmids using Lipofectamine 2000 (Life Technologies). After 48 h, immunoprecipitation were performed using anti-FLAG M2 Affinity Gel (A2220, Sigma) or GFP-Trap _A agarose (gta-20, ChromoTek). FLAG-tagged or GFP-tagged proteins were detected by western blotting using anti-FLAG antibody (1:1000; F1804, Sigma) and anti-GFP antibody (1:10,000; ab290, Abcam).

### Western blotting

RIPA buffer (50 mM Tris-HCl, pH 7.4, 1% Nonidet P-40, 0.5% Na deoxycholate, 0.01% SDS, 1 mM EDTA, and 150 mM NaCl) was used to homogenize and lyse mouse testes. Testis protein lysates were separated by 4–20% SDS-PAGE gel and transferred to polyvinlylidene difluoride (PVDF) membranes (Bio-Rad). After blocking in 5% non-fat milk, the membranes were incubated with primary antibodies in blocking solution at 4 °C overnight. Membranes were washed with TBST for three times and incubated with HRP-conjugated goat anti-rabbit IgG (1:5000; 1706515, Bio-Rad) or goat anti-mouse IgG (1:5000; 1706516, Bio-Rad,) for 1 h. After rinsing with TBST for three times, chemiluminescent detection was performed. The primary antibodies used were: rabbit anti-TDRD5 (1:2000), mouse anti-β-actin (1:5000; A3854, Sigma), rabbit anti-MILI (1:2000; PM044, MBL), rabbit anti-MIWI (1:1000; 2079, Cell Signaling Technology). Uncropped versions of all blots are included as Supplementary Fig. 14.

### Immunoprecipitation of piRNAs

Mouse testes were homogenized in lysis buffer (20 mM HEPES, pH 7.3, 150 mM NaCl, 2.5 mM MgCl_2_, 0.2 % NP-40, and 1 mM DTT) with protease inhibitor cocktail (Thermo Scientific) and RNase inhibitor (Promega). The lysates were centrifuged at 13,000×*g* for 10 min after sonication. The supernatants were collected and pre-cleared with protein-A agarose beads (Roche) at 4 °C for 2 h. Anti-MILI (PM044, MBL) or anti-MIWI (ab12337, Abcam) antibodies were used for immunoprecipitation and protein-A agarose beads were added to the lysates and incubated for 4 h to capture immunocomplexes. The beads were then collected and washed in lysis buffer for five times. Immunoprecipited piRNAs were isolated using Trizol reagent (Thermo Scientific) for downstream piRNA labeling and small RNA library construction experiments.

### Detection of piRNAs

Total RNA was isolated from mouse testes using Trizol reagent (Thermo Scientific). Total RNA (1 μg) or immunoprecipitated RNA was de-phosphorylated with Shrimp Alkaline Phosphatase (NEB). RNA end-labeling was performed using T4 polynucleotide kinase (NEB) and [γ-^32^P] ATP. ^32^P-labeled RNA was separated on a 15% Urea-PAGE, and signals were detected by exposing the gel on phosphorimager screen. Images were obtained by scanning on the Typhoon scanner (GE Healthcare).

### Cell sorting

Pachytene spermatocytes were isolated using flow cytometry according to a published protocol with modifications^46^. Briefly, testes were collected from adult mice, and the tunica was removed. Testes were digested for 10 min at 32 °C in HBSS with 50 U ml^−1^ collagenase IV (Life Technologies, 17104–019). Seminiferous tubules were washed once with HBSS, and digested for 50 min at 32 °C in HBSS with 100 U ml^−1^ collagenase IV and 5 μg ml^−1^ DNase I (Sigma, DN-25). The cells were resuspended thoroughly and filtered with a 70 μm cell strainer. Single-cell suspension was stained in HBSS with 5% FBS and 10 μg ml^−1^ Hoechst 33342 (Thermo Scientific) for 10 min at 32 °C. Volume of 2 μg ml^−1^ propidium iodide (Sigma) was added to exclude dead cells. Cells were sorted using Influx FACS (BD Biosciences). Hoechst was excited with a UV laser at 355 nm, and fluorescence was recorded with a 460/50 filter (Hoechst Blue) and 670/30 filter (Hoechst Red).

### Small RNA libraries and bioinformatics

Immunoprecipitated RNAs or total RNA were used to construct small RNA libraries. Small RNA libraries were prepared using NEBNext Multiplex Small RNA Library Prep Kit (E7300, NEB). Multiple libraries with different barcodes were pooled together and sequenced using the Illumina HiSeq 2500 platform (MSU Genomic Core Facility). See Supplementary Table 4 for the list of small RNA libraries. Three biological repeats of total piRNA libraries and MILI bound piRNA libraries were constructed and sequenced using pairs of WT and *Tdrd5*^*cKO*^ mice. The data from a representative WT and *Tdrd5*^*cKO*^ library pair are shown.

*fastx_clipper* was used to process sequenced reads by clipping the sequencing adapter read-through. Clipped reads were filtered by length (24–32 nt, unless otherwise indicated). These reads were then aligned to 5 sets of sequences sequentially: (1) 214 piRNA clusters^19^, (2) coding RNAs (RefSeq coding gene mRNAs), (3) non-coding RNAs (Refseq non-coding gene mRNAs), (4) Repeats (LINE, SINE, LTR, DNA, Low_complexity, Satellite, Simple_repeat), and (5) Intron (Genic regions for RefSeq genes). For alignment to each sequence set, only sequence reads that were not aligned to any of the previous sets were included. Sequence reads not mapping to the above 5 sets of sequences were classified as ‘other’. Here we define “non-cluster” as all reads not mapping to the 214 piRNA clusters. It includes the sum of 5 categories: coding RNA, non-coding RNA, repeats, intron, and other. Alignments were performed using Bowtie (one base mismatch allowed). The Repeats sequence set used here is defined by RepeatMasker (http://hgdownload.cse.ucsc.edu/goldenPath/mm10/database/rmsk.txt.gz).

For total piRNA analyses, small RNA reads (24–32 nt) were normalized between paired WT and mutant libraries based on miRNA counts (21–23 nt). For analyses of MILI or MIWI bound piRNAs, small RNA reads (24–32 nt) were normalized based on the total reads of each library. Graphs for alignment depth were produced with GraphPad Prism.

### TDRD5 RNA immunoprecipitation

Testes from wild-type and *Tdrd5*^*cKO*^ mice were collected, detunicated, disrupted by mild pipetting in ice-cold HBSS, and immediately UV-irradiated three times at 400 mJ cm^−2^ in stratalinker (Stratagene). The cells were washed with PBS once and lysed by RIPA buffer containing protease inhibitors and RNase inhibitors. The lysates were pre-cleared using protein-A agarose beads. The pre-cleared lysates were incubated with 3 μg TDRD5 antibody or rabbit IgG as a control for 4 h at 4 °C. After incubating with protein-A agarose beads for 2 h at 4 °C, the beads were washed with RIPA buffer for five times. For RNA isolation, the beads were treated with DNase I for 10 min at 37 °C followed by treatment with proteinase K for 1 h at 65 °C with shaking. RNAs were isolated using Trizol. Isolated RNAs were reverse transcribed with iScript cDNA Synthesis Kit (Bio-Rad). RT-PCR was performed with the primers shown in Supplementary Table 2.

### TDRD5 HITS-CLIP

TDRD5 HITS-CLIP was performed as previously described with modification^21,44,47^. Briefly, testes from adult mice were detunicated and disrupted by pipetting in ice-cold HBSS. Two testes were used for each HITS-CLIP. Tissue suspension was immediately UV-irradiated three times at 400 mJ cm^−2^ in Stratalinker UV crosslinker (Stratagene) with 30 s intervals for cooling. The UV treated cells were pelleted, washed in PBS, and lysed in 300 μl 1X PMPG buffer with protease inhibitors (Roche) and rRNasin (Promega). No exogenous nucleases were added into the lysis buffer. Lysates were treated with RQ1 DNase (Promega) for 5 min at 37 °C and centrifuged at 90,000×*g* for 30 min at 4 °C.

For each immunoprecipitation, TDRD5 antibody, MILI antibody (PM044, MBL), or Rabbit IgG (2729, Cell Signaling Technology) were bound on protein A Dynabeads (Life Technologies) in antibody binding buffer for 3 h at 4 °C. The beads were washed in antibody binding buffer, followed by 1X PMPG buffer, and incubated with lysates for 3 h at 4 °C. After washing^47^, the beads were treated with Antarctic phosphatase (NEB). The 3′ RNA linker ligation was performed on beads using ^32^P-labeled RL3 RNA linkers followed by T4 PNK (NEB) treatment.

RNA-protein complexes were eluted from immunoprecipitated beads using 30 μl loading buffer for 12 min at 70 °C. Samples were analyzed by Novex NuPAGE 4–12% Bis-Tris gel (Life Technologies). The RNA-protein complexes were transferred onto nitrocellulose membrane and exposed overnight. Nitrocellulose membrane fragments containing the main radioactive signal were cut. RNA was extracted from the membrane fragments followed by 5′ linker ligation. Reverse transcription was performed using DP3 primer (IDT). RT-PCR was performed with DP3 and DP5 primer. PCR products were reamplified (RE-PCR) with the modified DSFP3 and DSFP5 primers (Supplementary Table 3). cDNA from two PCR steps was resolved on and extracted from 3% Metaphor 1X TAE gels (Lonza) stained with SYBR Safe. DNA was extracted with QIAquick gel extraction kit (Qiagen) and analyzed by Illumina deep sequencing. The cDNA libraries were sequenced on an Illumina MiSeq at 300 cycles.

Sequenced reads were processed with *fastx_clipper* to clip the sequencing adapter read-through. Clipped reads were filtered by length (≥15 nt) and aligned to the following sets of sequences: 214 piRNA clusters, coding RNAs, non-coding RNAs, repeats, intron and other.

### Quantitative RT-PCR

Total RNA was extracted from mouse testes using Trizol. For cDNA synthesis, 1 μg of RNA was treated with DNase I (M0303S, NEB) and was reverse transcribed with iScript cDNA Synthesis Kit (Bio-Rad). Quantitative PCR was performed in triplicate wells using CFX96 Real-Time PCR detection system with SYBR Green SuperMix (Bio-Rad). Three biological replications were performed. GAPDH was used as a reference gene.

### Data availability

All sequencing data are deposited in the Sequence Read Archive of NCBI under the accession number SRP093845. All other data that support the findings of this study are available from the corresponding authors upon reasonable request.

## Electronic supplementary material

Supplementary Information
