# Supplementary material for: TDRD5 binds piRNA precursors and selectively enhances pachytene piRNA processing in mice
Source: Nat Commun. 2018 Jan 9;9:127. doi: 10.1038/s41467-017-02622-w (PMC5760656; doi:10.1038/s41467-017-02622-w)
Supplement: Supplementary file 1 — Supplementary Information [file 41467_2017_2622_MOESM1_ESM.pdf]

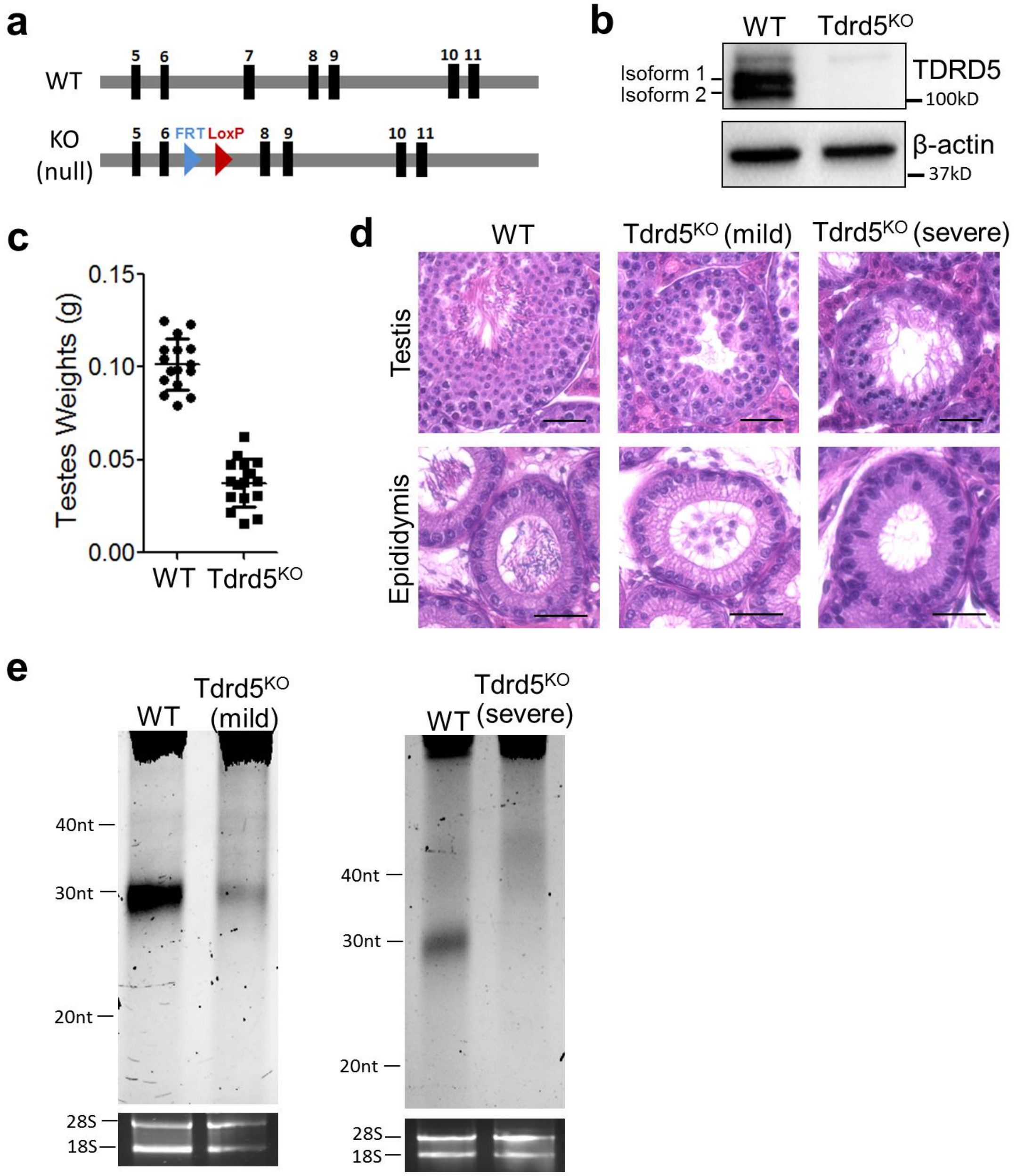

Supplementary Figure 1: Generation of *Tdrd5* global knockout mice. (**a**) A schematic diagram showing *Tdrd5* WT and null (KO) alleles. (**b**) Western blotting was performed with TDRD5 antibody using total testis lysates from adult wild-type (WT) or $Tdrd5^{KO}$ mice. The two TDRD5 bands represent isoform 1 and isoform 2 of TDRD5. (**c**) Testicular atrophy in $Tdrd5^{KO}$ mice. Testis weights of adult WT and $Tdrd5^{KO}$ littermates are shown. n=16. *Error bars* represent s.e.m. (**d**) Hematoxylin and eosin staining of testis and epididymis sections from adult WT, mild $Tdrd5^{KO}$ and severe $Tdrd5^{KO}$ mice. Spermatogenic arrest occurred at round spermatids stage in mild $Tdrd5^{KO}$ testes and at zygotene spermatocyte stage in severe $Tdrd5^{KO}$ testes. Scale bar, 40μm. (**e**) Reduced piRNA production in adult $Tdrd5^{KO}$ testes. Total piRNA levels from 10 μg of total RNA from WT, mild and severe $Tdrd5^{KO}$ testes and detected by 15% TBE urea gel. 28S and 18S rRNA was used as a loading control.

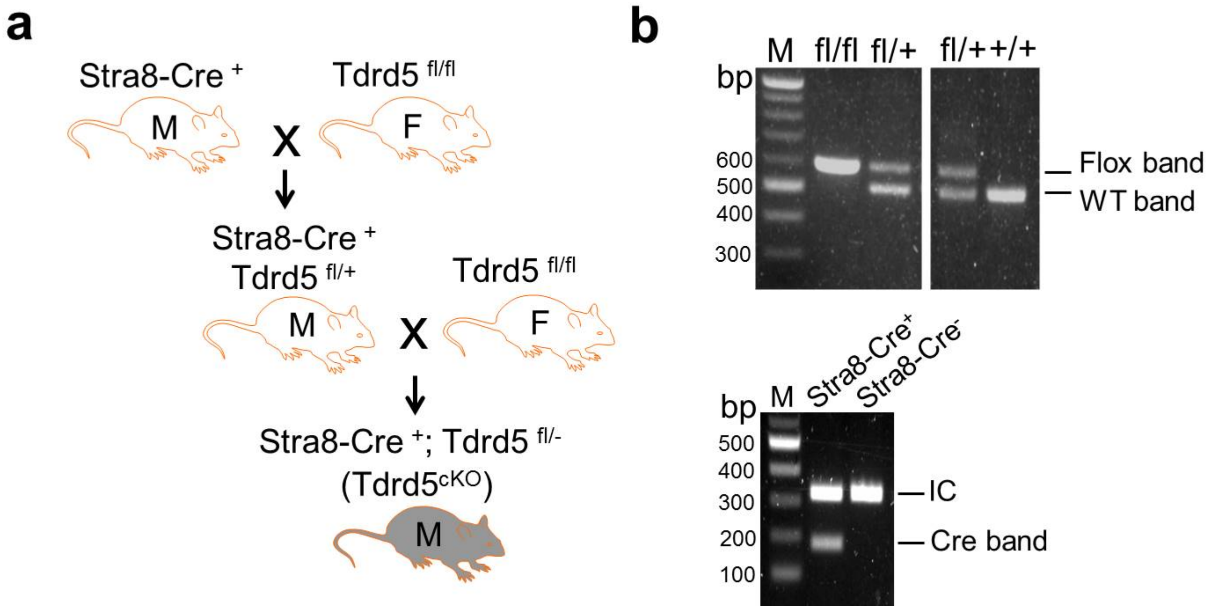

Supplementary Figure 2: Conditional inactivation of *Tdrd5* in postnatal male germ cells in mice. (**a**) The breeding scheme to generate Stra8-Cre $Tdrd5^{cKO}$ mice. The expression of Stra8-Cre at postnatal Day 3 resulted in Cre mediated deletion of *Tdrd5* in postnatal male germ cells. (**b**) Genotyping PCRs of $Tdrd5^{fl}$ allele and Stra8-Cre allele. An internal control (IC) PCR was used to indicate the presence of genomic DNA.

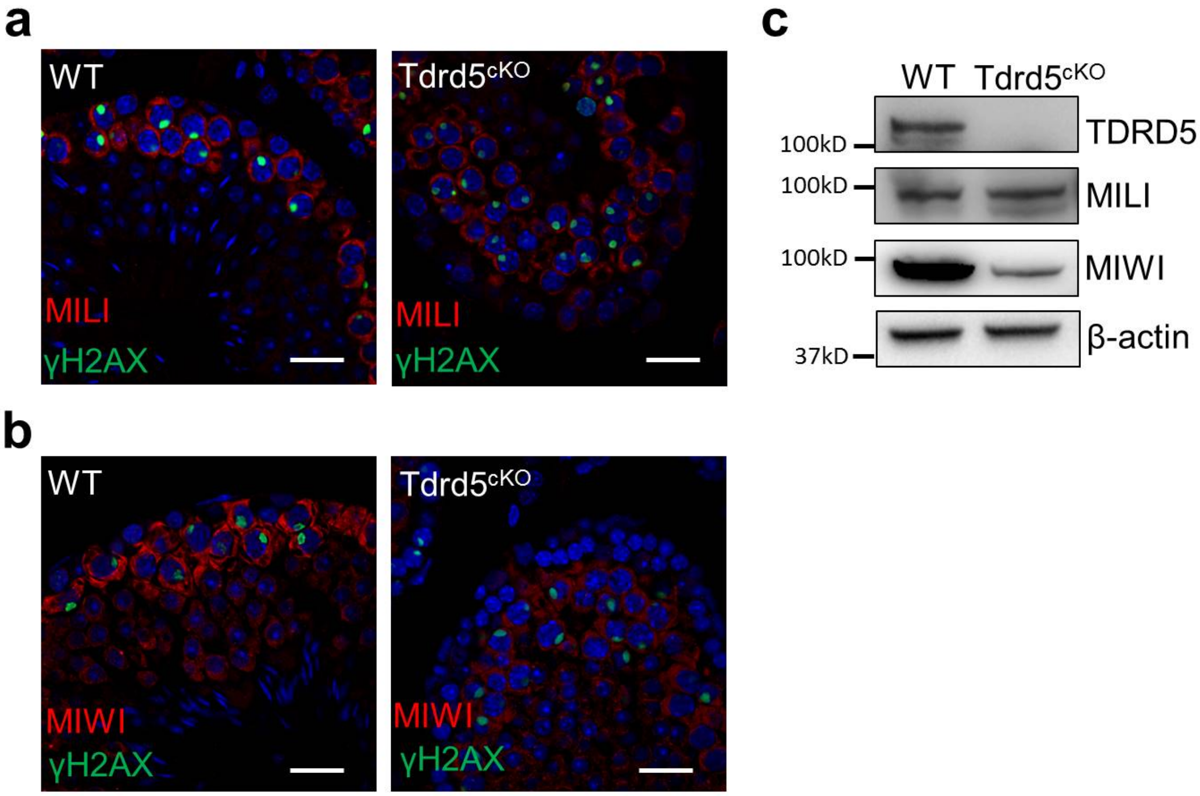

Supplementary Figure 3: Expression and localization of MILI and MIWI in adult wild-type and *Tdrd5^{cKO}* testes. (**a**, **b**) Co-immunostaining of MILI and γH2AX (A) or MIWI and γH2AX (B) in WT and *Tdrd5^{cKO}* testes. DNA was stained by DAPI. γH2AX staining in XY bodies labels pachytene spermatocytes. The MILI and MIWI localization was globally unaffected in *Tdrd5^{cKO}* testes. Immunofluorescence intensity of MILI in *Tdrd5^{cKO}* was similar to that of WT. The MIWI expression level was decreased in *Tdrd5^{cKO}* testes. Scale bar, 20µm. (**c**) Western blotting with indicated antibodies on testis lysates from WT and *Tdrd5^{cKO}* adult testes. MIWI protein expression was decreased while MILI expression was unaffected in *Tdrd5^{cKO}* testes.

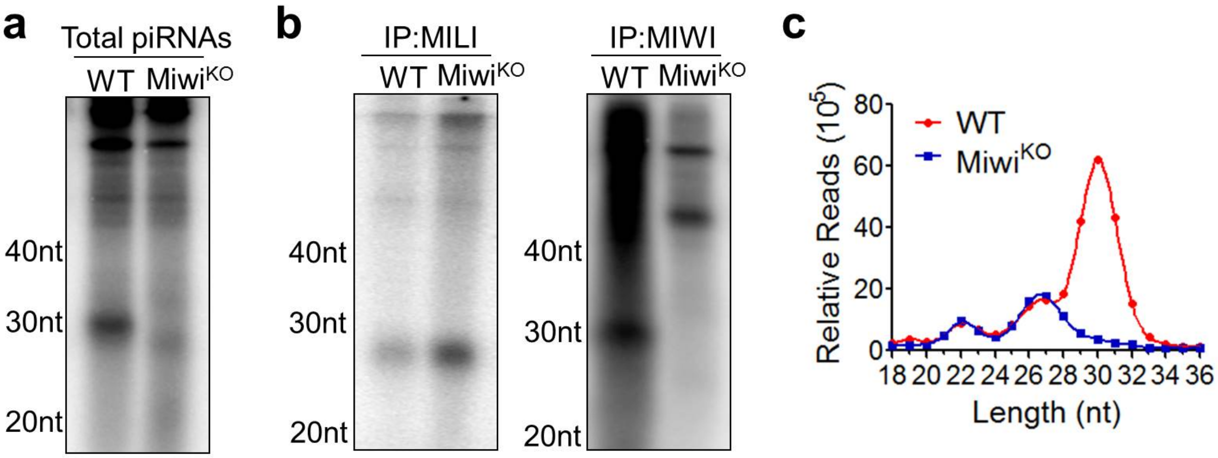

Supplementary Figure 4: piRNAs in adult *Miwi*^{KO} mice. (**a**) Total RNAs from adult WT and *Miwi*^{KO} testes were end-labeled with [^{32}P]-ATP and detected by denaturing PAGE. The piRNA expression level was significantly decreased in *Miwi*^{KO} mice. (**b**) Immunoprecipitated MILI-piRNAs and MIWI-piRNAs from adult WT and *Miwi*^{KO} were end-labeled with [^{32}P]-ATP and detected by denaturing PAGE. MIWI-piRNAs were absent in *Miwi*^{KO} testes due to lack of MIWI proteins. (**c**) Length distribution of total small RNAs from adult WT and *Miwi*^{KO} testes. Small RNA libraries were constructed using total RNA and sequenced. The curves of RNA reads were normalized by miRNA reads (21nt-23nt) from each library.

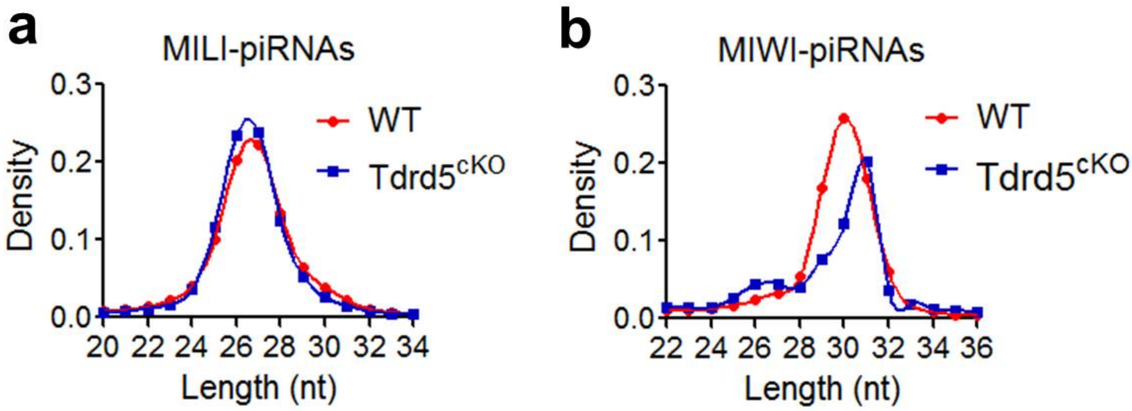

Supplementary Figure 5: The length distribution of MILI-piRNAs and MIWI-piRNAs in wild-type and *Tdrd5^{cKO}* testes. (**a**) The length distribution of MILI-piRNAs from adult WT and *Tdrd5^{cKO}* MILI-piRNA libraries. (**b**) The length distribution of MIWI-piRNAs from adult WT and *Tdrd5^{cKO}* MIWI-piRNA libraries.

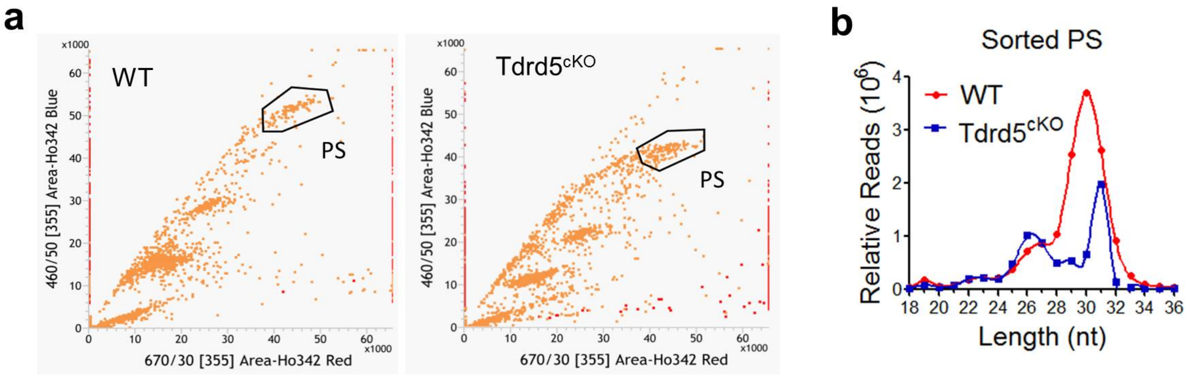

Supplementary Figure 6: piRNA distribution in sorted pachytene spermatocytes. (**a**) Pachytene spermatocytes (PS) were sorted from adult WT and *Tdrd5*^{cKO} testes using Hoechst 33342 and propidium iodide. (**b**) Length distribution of total small RNAs from WT and *Tdrd5*^{cKO} PS. Data were normalized using miRNA reads.

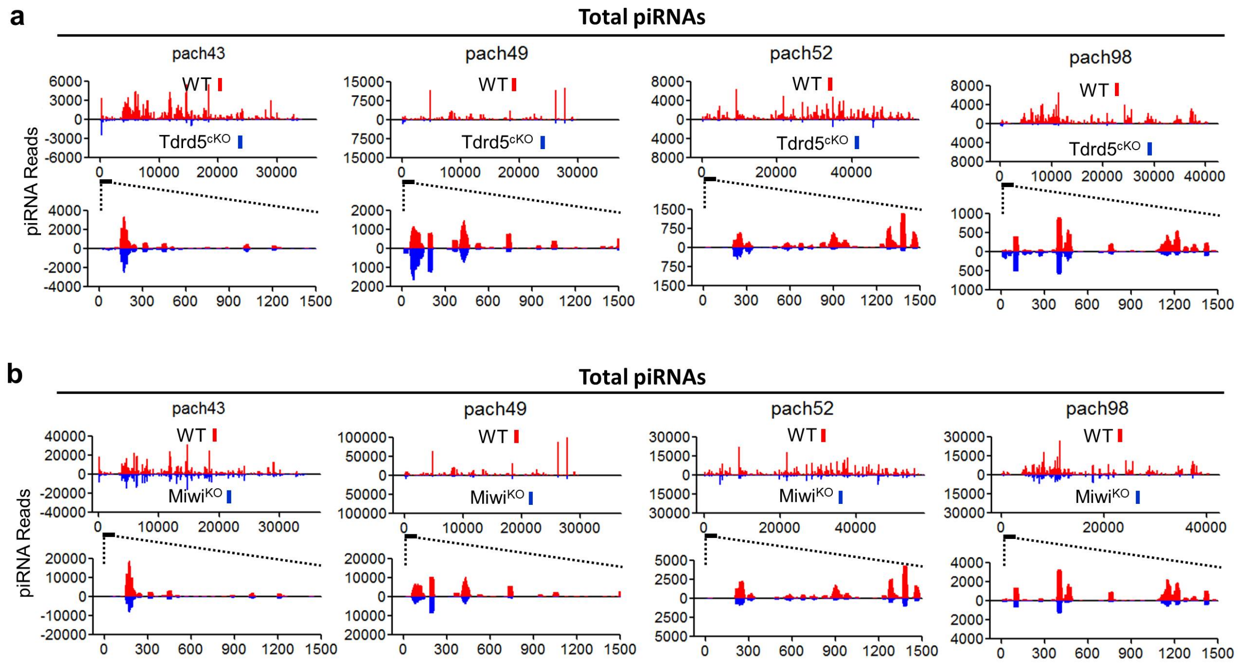

Supplementary Figure 7: Normal and zoomed view of total piRNA reads from wild-type and *Tdrd5^{cKO}* testes mapping to representative piRNA clusters. (**a**) Mapping of total piRNAs (24-32 nt) from WT (red) and *Tdrd5^{cKO}* (blue) testes to piRNA clusters 43, 49, 52 and 98. Data were normalized by miRNA (21-23 nt) counts. The 5' region including the first 1500 nt from each precursor transcripts is zoomed. (**b**) Mapping of total piRNAs (24-32 nt) from WT (red) and *Miwi^{KO}* (blue) testes to piRNA clusters 43, 49, 52 and 98. Data were normalized by miRNA (21-23 nt) counts. The 5' region including the first 1500 nt from each precursor transcripts is zoomed.

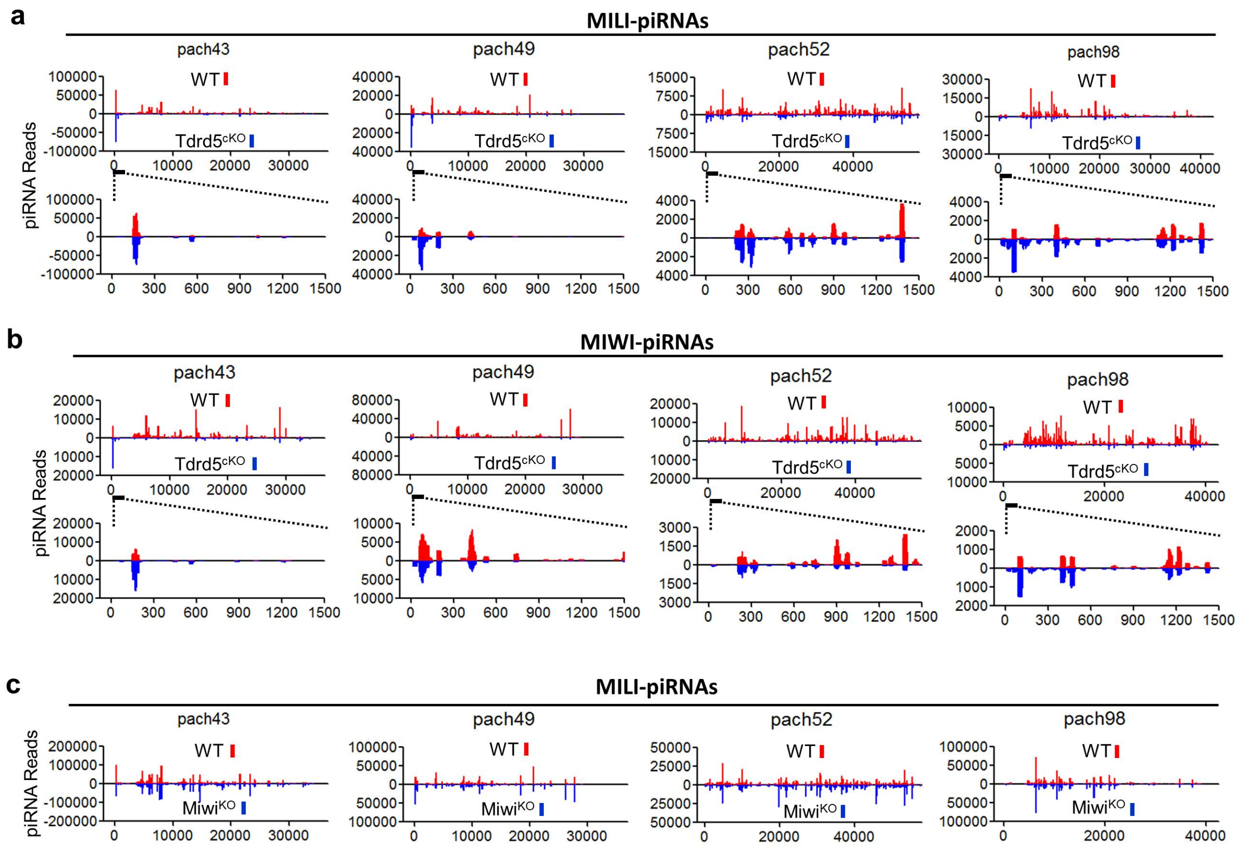

Supplementary Figure 8: Normal and zoomed view of MILI-bound or MIWI-bound piRNA reads from wild-type and *Tdrd5*^{cKO} testes mapping to representative piRNA clusters. (**a**) Mapping of MILI-piRNAs from WT (red) and *Tdrd5*^{cKO} (blue) mice to piRNA clusters 43, 49, 52 and 98. The 5' region including the first 1500 nt from each precursor transcripts is zoomed. (**b**) Mapping of MIWI-piRNAs from WT (red) and *Tdrd5*^{cKO} (blue) mice to piRNA clusters 43, 49, 52 and 98. The 5' region including the first 1500 nt from each precursor transcripts is zoomed. (**c**) Mapping of MILI-piRNAs from WT (red) and *Miwi*^{KO} (blue) mice to piRNA clusters 43, 49, 52 and 98.

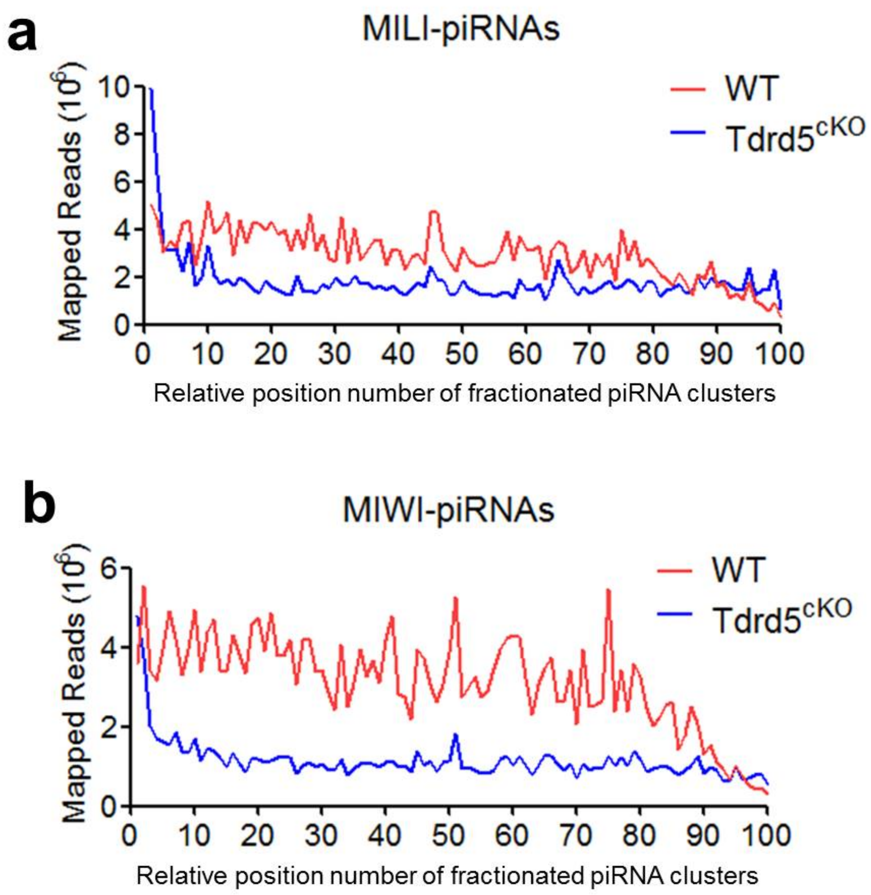

Supplementary Figure 9: TDRD5 deficiency does not cause reduction in piRNA production from 5' ends within single piRNA clusters. (**a**) MILI-piRNAs from WT and *Tdrd5*^{cKO} libraries were mapped onto 214 piRNA clusters. The density plots of mapped piRNA reads at relative positions of 214 piRNA clusters were shown. Data were normalized by total small RNA reads from each library pair. (**b**) MIWI-piRNAs from WT and *Tdrd5*^{cKO} libraries were mapped onto 214 piRNA clusters. The density plots of mapped piRNA reads at relative positions of 214 piRNA clusters were shown. Data were normalized by total small RNA reads from each library pair.

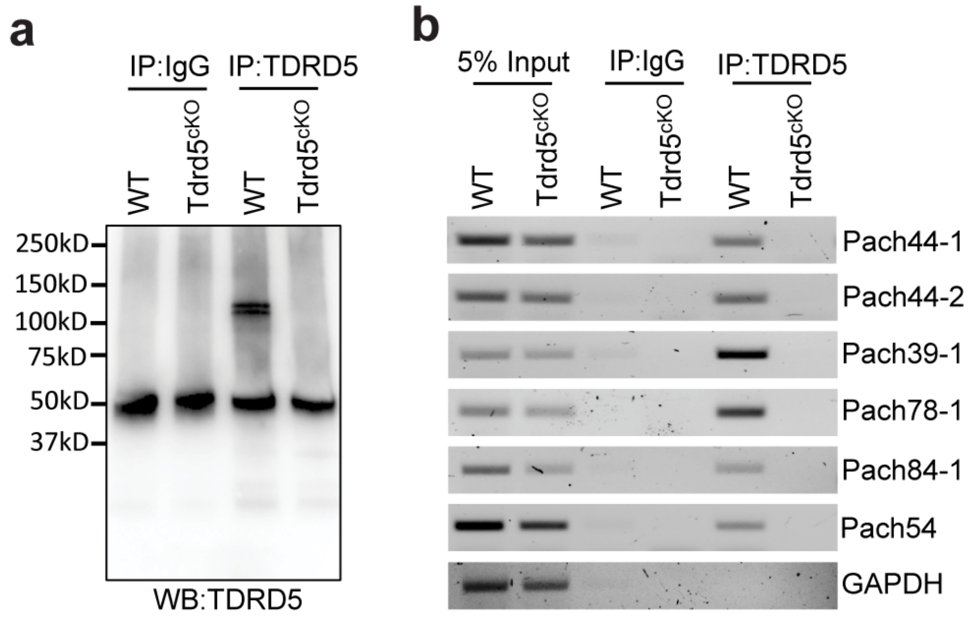

Supplementary Figure 10: UV cross-linking RNA immunoprecipitation of TDRD5 from adult wild-type and *Tdrd5^{cKO}* testes. (**a**) Western blotting showing the efficiency and specificity of TDRD5 antibody. (**b**) RT-PCR showing the specific presence of piRNA precursors in TDRD5 immunoprecipitates. GAPDH served as a negative control.

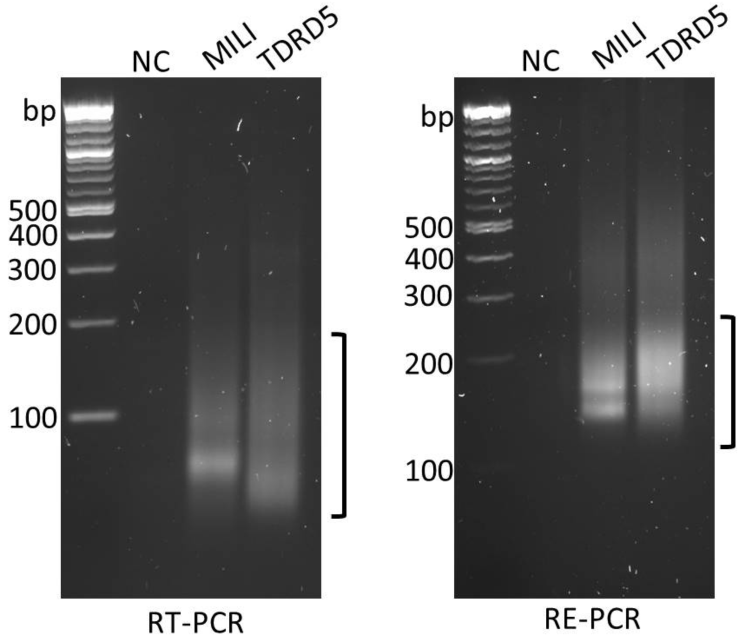

Supplementary Figure 11: RT-PCR and PCR reamplification of TDRD5 and MILI crosslinked RNA. PCR products were gel purified after Metaphor agarose gel electrophoresis. Black brackets indicate the isolated DNA regions.

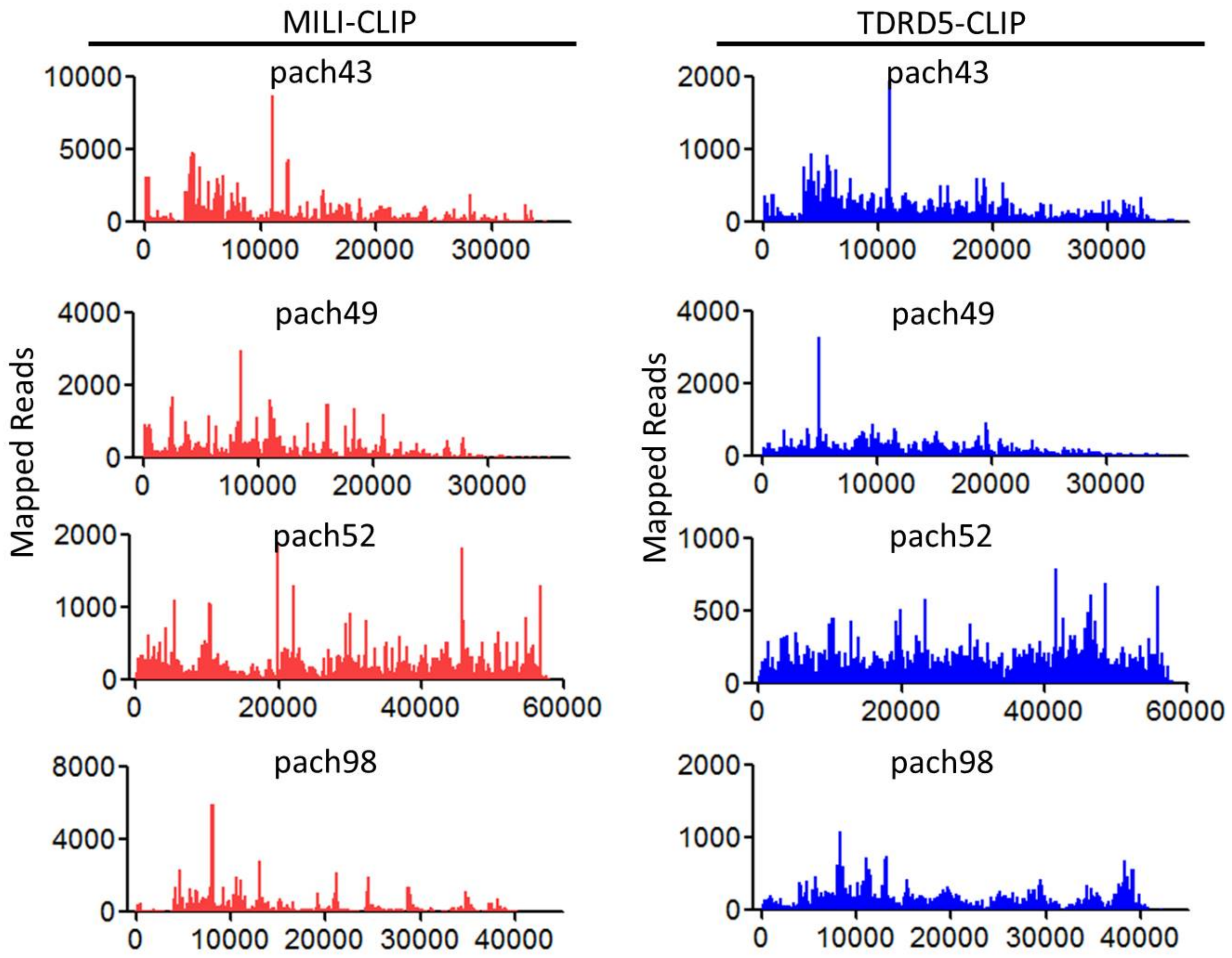

Supplementary Figure 12: TDRD5 binds piRNA precursors across their entire lengths. Mapping of MILI-CLIP and TDRD5-CLIP reads to piRNA clusters 43, 49, 52 and 98.

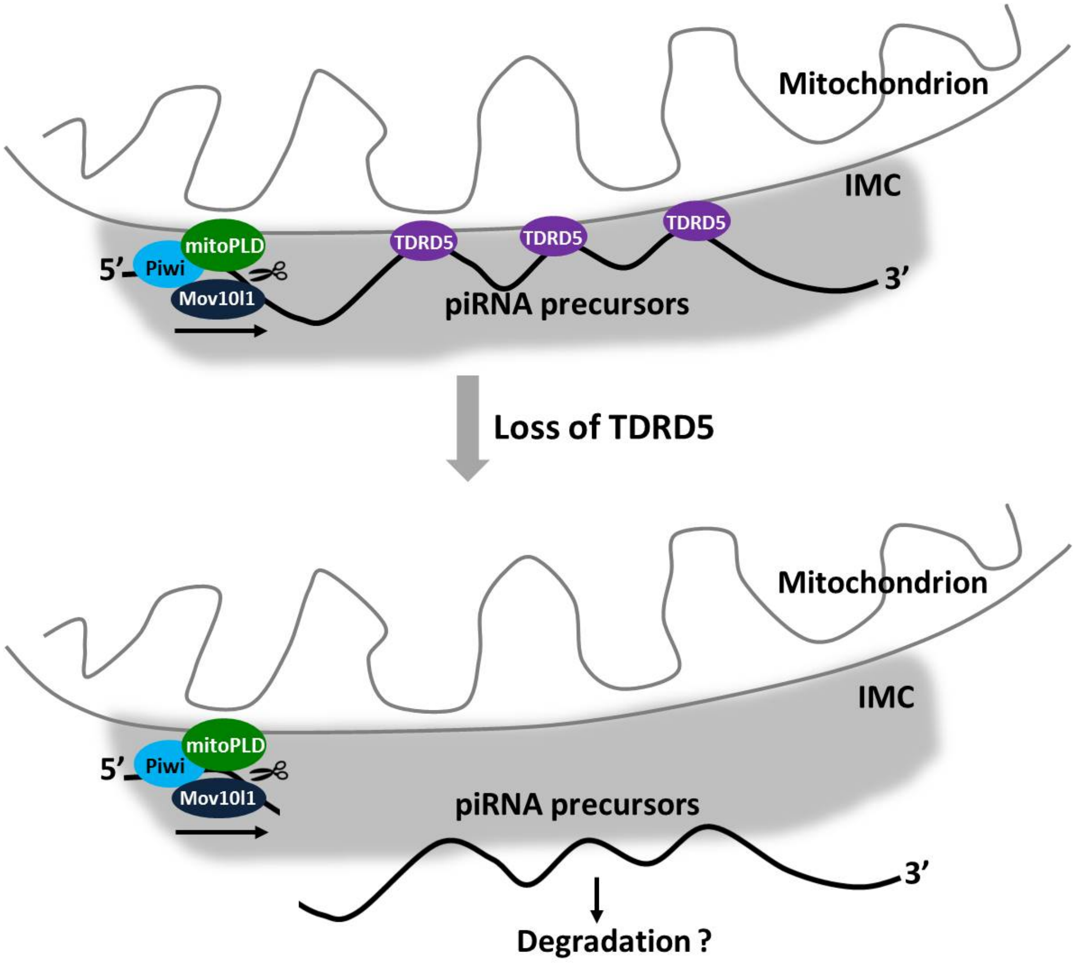

Supplementary Figure 13: **A model illustrating the role of TDRD5 in pachytene piRNA precursor processing.** piRNA precursors transcribed from piRNA clusters are transported from the nucleus and enter into the piRNA processing complex in intermitochondrial cement (IMC) by unknown mechanism. TDRD5 directly interacts with piRNA precursors and guides the selection and efficiency of piRNA processing. TDRD5 may retain and stabilize piRNA precursors, and promote the processivity of piRNA processing within single piRNA precursors following precursor 5' end processing.

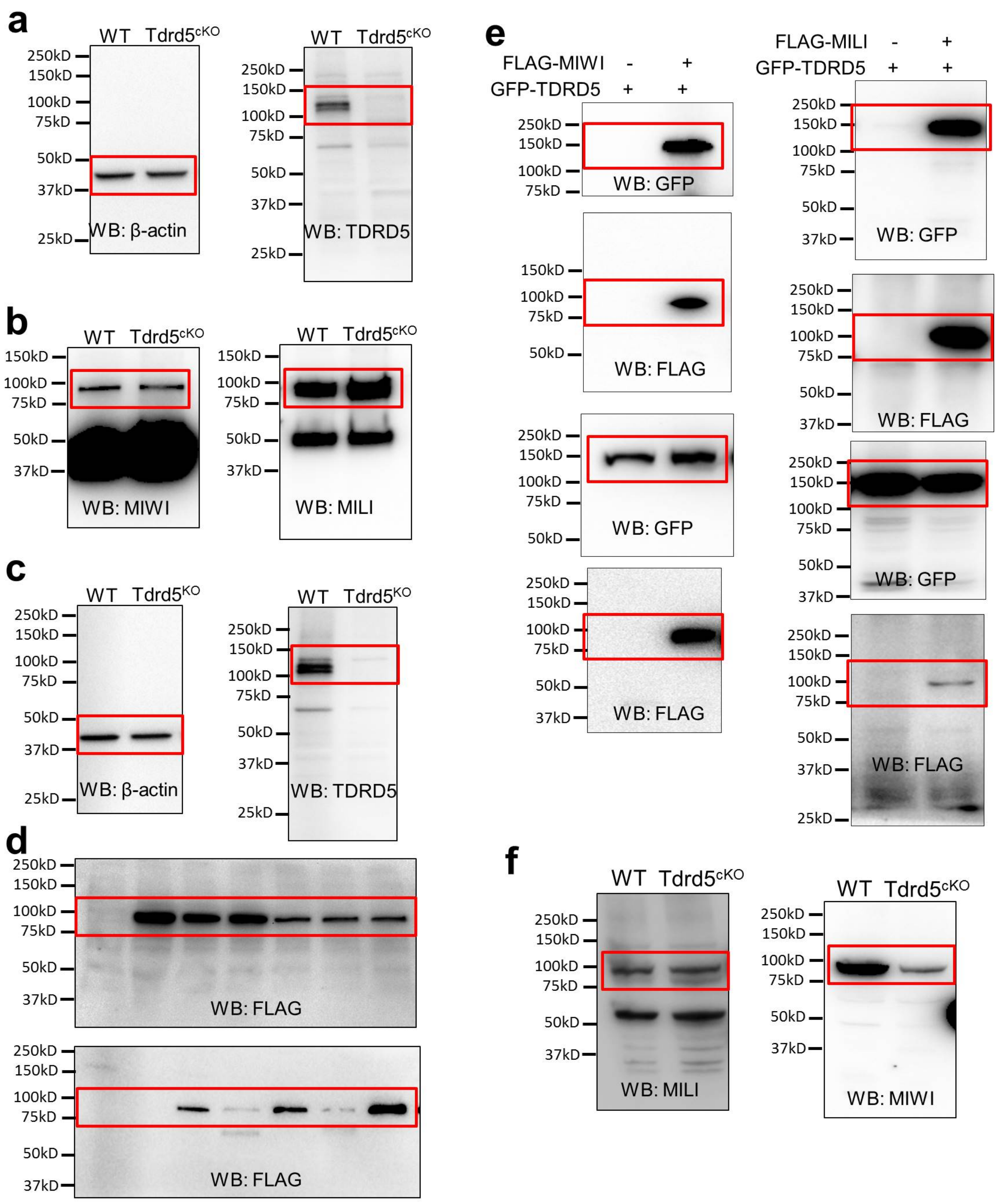

Supplementary Figure 14: Original Western blots shown in the manuscript. (**a**) Western blots correspond to Figure 1c. (**b**) Western blots correspond to Figure 2c. (**c**) Western blots correspond to Supplementary Figure 1b. (**d**) Western blots correspond to Figure 6b. (**e**) Western blots correspond to Figure 6a. (**f**) Western blots correspond to Supplementary Figure 3c.

Supplementary Table 1: Information on 214 piRNA clusters (mouse genome browser-mm9 assembly).

Name	Other Name	Chr	Start	Stop	Strand	Length
pach1	1-qC1.3-637.1	chr1	57405819	57434364	-	12274
pach2	1-qD-2017.1	chr1	93301458	93302606	-	544
pach3	1-qD-4525.1	chr1	94888921	94893644	-	4723
pach4	1-qE3-706.1	chr1	127071468	127074556	+	3088
pach5	10-qA3-2592.1	chr10	18516611	18551736	-	2082
pach6	10-qA3-143.1	chr10	20030311	20032118	-	1807
pach7	10-qB4-6488.1	chr10	62114440	62164257	+	49817
pach8	10-qB5.1-5404.1	chr10	66154778	66160884	-	6106
pach9	10-qB5.1-221.1	chr10	66161040	66171440	+	10400
pach10	10-qC1-12816.1	chr10	75300268	75324443	+	24175
pach11	10-qC1-117.1	chr10	83951038	83967582	+	16544
pach12	10-qC1-2617.1	chr10	85211306	85238346	+	27040
pach13	10-qC1-1527.1	chr10	86011423	86054254	-	42831
pach14	10-qC1-875.1	chr10	86079756	86088620	+	8864
pach15	10-qC2-545.1	chr10	94136457	94151187	-	3744
pach16	pi-Tmem194.1	chr10	127114107	127132221	+	1531
pach17	11-qB1.3-590.1	chr11	50755203	50757227	-	2024
pach18	11-qE1-9443.1	chr11	103286577	103315010	+	28433
pach19	11-qE1-3997.1	chr11	107855325	107859037	+	3439
pach20	11-qE1-252.1	chr11	108278889	108286739	-	7850
pach21	12-qE-23911.1	chr12	99620581	99658258	-	36814
pach22	12-qE-7089.1	chr12	99658453	99692927	+	34474
pach23	13-qA3.1-213.1	chr13	21595489	21598393	-	1629
pach24	13-qA3.1-355.1	chr13	24997468	25026901	+	771
pach25	13-qA5-208.1	chr13	50363393	50412729	+	31835
pach26	13-qA5-464.1	chr13	50607591	50690856	-	12716
pach27	13-qA5-703.1	chr13	51001008	51029517	-	9379
pach28	13-qA5-967.1	chr13	52192103	52219527	+	4028
pach29	13-qB1-1517.1	chr13	53489036	53549907	+	1268
pach30	14-qA3-3095.1	chr14	20445381	20472632	-	27251
pach31	14-qA3-19970.1	chr14	24901215	24939690	+	38475
pach32	14-qA3-2286.1	chr14	25184829	25189036	-	4207
pach33	14-qA3-284.1	chr14	25244385	25249047	-	4662
pach34	14-qC1-1261.1	chr14	45377787	45409614	-	31827
pach35	14-qC1-1010.1	chr14	45546497	45569941	+	18625
pach36	15-qD1-17920.1	chr15	59081442	59106777	-	25335
pach37	15-qD1-4001.1	chr15	59106921	59123501	+	556
pach38	pi-1700016M24Rik.1	chr15	74436458	74466677	-	3477

pach39	15-qD3-14639.1	chr15	74466817	74478882	+	12065
pach40	15-qE1-8387.1	chr15	78483658	78500962	-	17304
pach41	15-qE1-1119.1	chr15	79758435	79764840	+	6405
pach42	17-qA3.3-352.1	chr17	22634368	22656090	+	6847
pach43	17-qA3.3-27363.1	chr17	27425220	27461973	-	36753
pach44	17-qA3.3-26735.1	chr17	27462141	27504428	+	38339
pach45	17-qC-935.1	chr17	49251595	49252836	-	657
pach46	17-qC-59.1	chr17	50378485	50382342	+	3857
pach47	17-qE1.1-7037.1	chr17	66556151	66581098	+	24947
pach48	pi-Cdc42ep3.1	chr17	79734018	79754230	-	1112
pach49	18-qE1-36451.1	chr18	67189100	67226114	-	37014
pach50	18-qE1-1295.1	chr18	67226241	67241315	+	15074
pach51	19-qC2-1361.1	chr19	37333596	37338356	-	2149
pach52	2-qE1-35981.1	chr2	92381298	92439234	+	57936
pach53	2-qF1-2536.1	chr2	127517589	127529447	+	5394
pach54	2-qG3-1029.1	chr2	150953183	150984330	+	11705
pach55	3-qA2-617.1	chr3	20301593	20405121	-	67058
pach56	3-qA3-2052.1	chr3	34725552	34777871	+	6403
pach57	pi-1700006A11Rik.1	chr3	124103907	124128909	-	1932
pach58	4-qB3-3994.1	chr4	57373062	57377138	-	1558
pach59	4-qB3-639.1	chr4	61881631	61891970	-	8699
pach60	4-qB3-277.1	chr4	61892039	61900375	+	6673
pach61	4-qC5-17839.1	chr4	93946842	93998314	-	51472
pach62	4-qD2.2-2182.1	chr4	123510867	123519209	-	8342
pach63	4-qD2.2-349.1	chr4	123571373	123573843	-	2470
pach64	4-qD3-2082.1	chr4	135182710	135186113	+	2693
pach65	pi-Wdfy3.1	chr5	102261978	102280532	-	4690
pach66	5-qF-14508.1	chr5	113752221	113769115	-	16894
pach67	5-qF-14224.1	chr5	113769157	113794752	+	25537
pach68	5-qF-4633.1	chr5	115284179	115303596	-	19417
pach69	5-qG2-950.1	chr5	137395015	137412982	+	3973
pach70	5-qG2-2301.1	chr5	144519247	144527999	+	3868
pach71	5-qG3-23659.1	chr5	150592651	150627915	-	35264
pach72	6-qC3-6258.1	chr6	81843811	81860488	-	7342
pach73	6-qC3-100.1	chr6	83525934	83538118	+	3295
pach74	pi-Gm5878.1	chr6	85061409	85076088	-	4643
pach75	6-qC3-2394.1	chr6	85937105	85953600	-	4854
pach76	6-qD1-2831.1	chr6	87932334	87944161	-	11827
pach77	6-qF3-8009.1	chr6	127726093	127746390	-	18441
pach78	6-qF3-28913.1	chr6	127746448	127791908	+	45460
pach79	7-qB5-6255.1	chr7	60142976	60169237	+	7825

pach80	7-qD1-9417.1	chr7	77019095	77054469	-	35374
pach81	7-qD1-16444.1	chr7	77054649	77111245	+	54881
pach82	7-qD1-654.1	chr7	80242711	80250159	-	5208
pach83	7-qD1-19431.1	chr7	80250197	80271441	+	21244
pach84	7-qD2-24830.1	chr7	80926316	80961355	-	35039
pach85	7-qD2-11976.1	chr7	80961480	80977906	+	10203
pach86	7-qF3-3125.1	chr7	132476266	132493286	-	691
pach87	7-qF3-246.1	chr7	132493384	132508334	+	14950
pach88	8-qA2-343.1	chr8	28403548	28406760	-	3212
pach89	8-qA4-332.1	chr8	38155119	38158009	-	2890
pach90	8-qA4-155.1	chr8	38166951	38168562	-	1611
pach91	8-qC5-8200.1	chr8	94713358	94718315	+	4957
pach92	8-qC5-2209.1	chr8	95933840	95951276	-	14448
pach93	8-qE1-3748.1	chr8	112641565	112656356	+	638
pach94	9-qA1-178.1	chr9	3184709	3199792	-	6920
pach95	pi-Arhgap20.1	chr9	51573456	51661164	+	6364
pach96	9-qA5.3-24188.1	chr9	54054980	54097630	-	42650
pach97	9-qA5.3-1495.1	chr9	54097752	54117106	+	19354
pach98	9-qC-31469.1	chr9	67539058	67581593	-	42535
pach99	9-qC-10667.1	chr9	67581751	67608736	+	26985
pach100	9-qF4-150.1	chr9	122711578	122714587	-	3009
hybrid1	pi-Asb1.1	chr1	93437656	93456160	+	5765
hybrid2	pi-Rc3h1.1	chr1	162836514	162905104	+	11035
hybrid3	pi-Eif4ebp2.1	chr10	60891239	60915546	-	5920
hybrid4	pi-Ipmk.1	chr10	70807660	70848629	+	8293
hybrid5	pi-Zfp280b.1	chr10	75495400	75505714	+	4830
hybrid6	pi-Klhl11.1	chr11	100318438	100334096	-	3383
hybrid7	pi-Ccdc117.1	chr11	5428892	5442204	-	9686
hybrid8	pi-Zfp652.1	chr11	95610456	95633664	+	7923
hybrid9	pi-Mrs2.1	chr13	25079352	25112173	-	6959
hybrid10	13-qA5-156.1	chr13	49910090	49991061	+	13377
hybrid11	pi-Eif2c2.1	chr15	72926278	73015616	-	14046
hybrid12	pi-Mlc1.1	chr15	88786320	88813107	-	5200
hybrid13	pi-Pou6f1.1	chr15	100405749	100410665	-	6323
hybrid14	15-qE1-3710.1	chr15	83184277	83197710	-	3659
hybrid15	pi-Strbp.1	chr2	37425396	37558890	-	16518
hybrid16	pi-Zmat3.1	chr3	32233715	32264587	-	7782
hybrid17	pi-BC026590.1	chr4	56815451	56822469	+	4040
hybrid18	pi-Kctd7.1	chr5	130620791	130630984	+	3538
hybrid19	pi-Tbl2.1	chr5	135625562	135638536	+	4358
hybrid20	pi-Mgll.1	chr6	88674463	88775841	+	1105

hybrid21	pi-Nr2c2.1	chr6	92041395	92123051	+	7635
hybrid22	6-qF3-3040.1	chr6	128122114	128150600	-	8130
hybrid23	6-qF3-1063.1	chr6	128388817	128456925	+	5072
hybrid24	pi-Zfp382.1	chr7	30906942	30925766	+	7932
hybrid25	pi-Tacc1.1	chr8	26266101	26314352	-	5180
hybrid26	pi-Tktl2.1	chr8	69035636	69043098	+	6370
hybrid27	pi-Ddx19b.1	chr8	113527086	113555623	-	6548
hybrid28	pi-Exoc8.1	chr8	127417011	127421676	-	4665
hybrid29	pi-Cbl.1	chr9	43951061	44041585	-	11161
hybrid30	pi-Ip6k1.1	chr9	107904975	107951111	+	4413
prepach1	pi-Fam168b.1	chr1	34870071	34899867	-	5007
prepach2	pi-Hjurp.1	chr1	90159688	90174093	-	4120
prepach3	pi-Ing5.1	chr1	95700553	95718679	+	4528
prepach4	1-qE2.3-2.1	chr1	120355721	120355843	-	122
prepach5	pi-Tfcp2l1.1	chr1	120524521	120581739	+	9264
prepach6	pi-Elk4.1	chr1	133904194	133928161	+	9558
prepach7	pi-Ppp1r15b.1	chr1	135027714	135036359	+	5598
prepach8	pi-Ppp1r12b.1	chr1	136651241	136852527	-	8843
prepach9	pi-Abl2.1	chr1	158488203	158579750	+	11116
prepach10	pi-Zbtb37.1	chr1	162933052	162964958	-	8800
prepach11	pi-Uhmk1.1	chr1	172123561	172145541	-	7866
prepach12	pi-Foxo3.1	chr10	41901651	41911816	-	4610
prepach13	pi-D10Wsu102e.1	chr10	82822985	82831579	+	3488
prepach14	pi-Ctdsp2.1	chr10	126415753	126437016	+	4827
prepach15	pi-Smcr8.1	chr11	60591039	60597792	+	3381
prepach16	pi-Lsmd1.1	chr11	69209318	69210176	+	495
prepach17	pi-Slc43a2.1	chr11	75345218	75391069	+	6901
prepach18	pi-Rab11fip4.1	chr11	79474214	79511524	+	7088
prepach19	pi-Igf2bp1.1	chr11	95818479	95868022	-	9143
prepach20	pi-Socs7.1	chr11	97223641	97259855	+	7429
prepach21	pi-Mllt6.1	chr11	97524530	97546757	+	7451
prepach22	pi-Wipf2.1	chr11	98724946	98764609	+	5271
prepach23	pi-Dcaf7.1	chr11	105898192	105920636	+	5741
prepach24	pi-Mafg.1	chr11	120486441	120495268	-	5266
prepach25	pi-Dnmt3a.1	chr12	3891106	3914443	+	6061
prepach26	pi-Map3k9.1	chr12	82815946	82882157	-	10609
prepach27	pi-Hist1h1a.1	chr13	23855536	23856215	+	679
prepach28	pi-Zfp346.1	chr13	55206682	55236190	+	3342
prepach29	pi-Nsd1.1	chr13	55313417	55419685	+	12035
prepach30	pi-Il17rd.1	chr14	27852218	27920472	+	8188
prepach31	pi-Hmbox1.1	chr14	65430438	65568699	-	9965

prepach32	pi-Tef.1	chr15	81633147	81657289	+	4102
prepach33	pi-Shank3.1	chr15	89331804	89390691	+	6982
prepach34	pi-Cbx5.1	chr15	103021983	103070259	-	8806
prepach35	pi-Ppm1f.1	chr16	16896549	16927451	+	4928
prepach36	pi-Hic2.1	chr16	17233679	17263523	+	6339
prepach37	pi-Crkl.1	chr16	17452059	17486929	+	4464
prepach38	pi-Lpp.1	chr16	24393531	24992661	+	15550
prepach39	pi-Zdhhc23.1	chr16	43964878	43979143	-	5638
prepach40	pi-Cramp1l.1	chr17	25098236	25152532	-	7407
prepach41	pi-Uhrf1bp1.1	chr17	27993451	28036985	+	8567
prepach42	pi-Kcng3.1	chr17	83973363	84031786	-	15840
prepach43	pi-Ndst1.1	chr18	60844148	60908308	-	7950
prepach44	pi-Fth1.1	chr19	10057193	10059582	+	865
prepach45	pi-Hif1an.1	chr19	44637337	44650762	+	6194
prepach46	pi-Gtf3c4.1	chr2	28677821	28695861	-	6999
prepach47	2-qE5-4.1	chr2	120518324	120540873	+	13441
prepach48	pi-Cbfa2t2.1	chr2	154262219	154365092	+	6080
prepach49	pi-Phf20.1	chr2	156022393	156135687	+	5720
prepach50	pi-Ccrn4l.1	chr3	51028854	51055547	+	2554
prepach51	pi-Gabpb2.1	chr3	94985683	95021902	-	8632
prepach52	pi-Mcart1.1	chr4	45411294	45421633	-	1933
prepach53	pi-D730040F13Rik.1	chr4	56879897	56960355	-	15901
prepach54	pi-Snx30.1	chr4	59818521	59917612	+	7507
prepach55	pi-Zyg11a.1	chr4	107847846	107890527	-	8981
prepach56	pi-Zyg11b.1	chr4	107900359	107973695	-	10654
prepach57	pi-Eya3.1	chr4	132195002	132280676	+	5114
prepach58	pi-Rcan3.1	chr4	134968222	134989706	-	5049
prepach59	pi-Luzp1.1	chr4	136025678	136110697	+	4125
prepach60	pi-Zbtb49.1	chr5	38591490	38611628	-	2738
prepach61	pi-Bend4.1	chr5	67783388	67819359	-	8005
prepach62	pi-Ssh1.1	chr5	114387108	114443767	-	8294
prepach63	pi-Mlec.1	chr5	115592990	115608225	-	5991
prepach64	pi-Fbxl18.1	chr5	143628624	143656891	-	6104
prepach65	pi-Tet3.1	chr6	83312367	83391602	-	10836
prepach66	pi-Fbxo41.1	chr6	85419571	85434653	-	6005
prepach67	pi-March08.1	chr6	116288039	116359551	+	4445
prepach68	pi-Bcl2l13.1	chr6	120786229	120842859	+	6950
prepach69	pi-Klf13.1	chr7	71031236	71083761	-	6356
prepach70	7-qD2-40.1	chr7	82913927	82926993	-	13066
prepach71	pi-Rnf169.1	chr7	107068766	107128968	-	7154
prepach72	pi-Fam53b.1	chr7	139903770	140044311	-	5812

prepach73	pi-Zfp866.1	chr8	72285224	72298794	-	5746
prepach74	pi-Cmtm4.1	chr8	106872110	106919708	-	7715
prepach75	pi-Atxn1l.1	chr8	112250549	112261649	-	7337
prepach76	pi-Gan.1	chr8	119682164	119739895	+	13331
prepach77	pi-Ankrd11.1	chr8	125406988	125566154	-	9422
prepach78	pi-Igsf9b.1	chr9	27148219	27165314	+	13620
prepach79	pi-Hinfp.1	chr9	44100521	44113717	-	5297
prepach80	pi-Rplp1.1	chr9	61761092	61762348	-	496
prepach81	pi-Rad54l2.1	chr9	106590412	106691503	-	9283
prepach82	pi-Trim71.1	chr9	114416339	114473487	-	9243
prepach83	pi-Acvr2b.1	chr9	119311403	119351032	+	10252
prepach84	pi-Exog.1	chr9	119354082	119373348	+	2637

Supplementary Table 2: Primer sequences for RT-PCR.

Primer Name	Sequence (5'-3')		PCR product length (bp)
pach44-2	F	AGTCTGTGTAGTAGTTTCCTGAG	109
	R	TGTCCACTTCCATGTTACCT	
pach39-1	F	GTTGCCCCAAGAGAATGTGT	200
	R	TTCCACAGGTCCAGCCTTAG	
pach84-1	F	CTCCCAATGGCAACATCTTT	199
	R	TGACCCTTCAGGGAATTCAG	
pach54	F	GATAACCACCAGCAGTTCTCCAC	133
	R	ACTCCTCATTGGTCCTTGTCTTG	
pach98	F	GTTAGCGAAGGACATTATTCTAACC	501
	R	TGACATGAACACAGGTGCTCAGAT	
pach44-1	F	ATTTCCTGGCTGGATGGTTGT	107
	R	AGGAGTGATGTGAGCGAGTGC	
pach67	F	CTATGCTTATGATGGCATTGGAGAG	530
	R	TTCCAGTTCAACAGGGACACGGGAC	
pach78-2	F	GTGAAGCTAAGGATGCTGGGATAG	413
	R	ACAGGATGTCCCCTGAAATCAGTC	
pri-let7g	F	GTACGGTGTGGACCTCATCA	137
	R	TCTTGCTGTGTCCAGGAAAG	
pach78-1	F	TGTTCACTTACATATCAGGGTC	118
	R	GTAAAGCCCAAGAGCAAGAG	
GAPDH	F	AGAAACCTGCCAAGTATGATGAC	167
	R	GTCATTGAGAGCAATGCCAG	

Supplementary Table 3: Primer sequences for HITS-CLIP seq.

Primer Name	Sequence (5'-3')
RL3 RNA oligo	GUGUCAGUCACUUCCAGCGG
RL5 RNA oligo	AGGGAGGACGAUGCGG
DP5	AGGGAGGACGATGCGG
DP3	CCGCTGGAAGTGACTGACAC
Modified DSFP5	AATGATACGGCGACCACCGAGATCTACACCTATGGATACTTAGTC AGGGAGGACGATGCGG
Modified DSFP3	CAAGCAGAAGACGGCATACGAGATCCGCTGGAAGTGACTGACAC
SSP1	CTATGGATACTTAGTCAGGGAGGACGATGCGG

Supplementary Table 4: Sequencing libraries used for analysis (available in the Sequence Read Archive with an accession number SRP093845).

Library Name
Stra8-cre Tdrd5 WT-1 total piRNA
Stra8-cre Tdrd5 cKO-1 total piRNA
Stra8-cre Tdrd5 WT-2 total piRNA
Stra8-cre Tdrd5 cKO-2 total piRNA
Stra8-cre Tdrd5 WT-3 total piRNA
Stra8-cre Tdrd5 cKO-3 total piRNA
Stra8-cre Tdrd5 WT-1 MILI-piRNA
Stra8-cre Tdrd5 cKO-1 MILI-piRNA
Stra8-cre Tdrd5 WT-2 MILI- piRNA
Stra8-cre Tdrd5 cKO-2 MILI-piRNA
Stra8-cre Tdrd5 WT-3 MILI-piRNA
Stra8-cre Tdrd5 cKO-3 MILI-piRNA
Stra8-cre Tdrd5 WT MIWI-piRNA
Stra8-cre Tdrd5 cKO MIWI-piRNA
Miwi WT total piRNA
Miwi KO total piRNA
Miwi WT MILI-piRNA
Miwi KO MILI-piRNA
Stra8-cre Tdrd5 WT sorted pachytene spermatocyte piRNA
Stra8-cre Tdrd5 cKO sorted pachytene spermatocyte piRNA
MILI-HITS-CLIP-Seq
TDRD5-HITS-CLIP-Seq-1
TDRD5-HITS-CLIP-Seq-2
TDRD5-HITS-CLIP-Seq-3
